# Evaluating the Impact of a Game (Inner Dragon) on User Engagement Within a Leading Smartphone App for Smoking Cessation: Randomized Controlled Trial

**DOI:** 10.2196/57839

**Published:** 2024-10-30

**Authors:** Justin S White, Séverine Toussaert, Bethany R Raiff, Marie K Salem, Amy Yunyu Chiang, David Crane, Edward Warrender, Courtney R Lyles, Lorien C Abroms, J Lee Westmaas, Johannes Thrul

**Affiliations:** 1 Department of Health Law, Policy and Management Boston University School of Public Health Boston, MA United States; 2 Philip R Lee Institute for Health Policy Studies University of California, San Francisco San Francisco, CA United States; 3 Department of Epidemiology and Biostatistics University of California, San Francisco San Francisco, CA United States; 4 Department of Economics University of Oxford Oxford United Kingdom; 5 Department of Psychology Rowan University Glassboro, NJ United States; 6 Department of Health Policy and Management Fielding School of Public Health University of California, Los Angeles Los Angeles, CA United States; 7 23 Limited London United Kingdom; 8 Department of Public Health Sciences University of California, Davis Davis, CA United States; 9 Department of Prevention and Community Health Milken Institute School of Public Health The George Washington University Washington, DC United States; 10 Population Science American Cancer Society Atlanta, GA United States; 11 Department of Mental Health Johns Hopkins Bloomberg School of Public Health Baltimore, MD United States; 12 Sidney Kimmel Comprehensive Cancer Center at Johns Hopkins Baltimore, MD United States; 13 Centre for Alcohol Policy Research La Trobe University Melbourne Australia

**Keywords:** smoking cessation, mobile app, games for health, gamification, engagement, randomized controlled trial, mobile phone

## Abstract

**Background:**

Smartphone apps are a convenient, low-cost approach to delivering smoking cessation support to large numbers of individuals. Yet, the apps are susceptible to low rates of user engagement and retention.

**Objective:**

This study aims to test the effects of a new game module (called Inner Dragon) integrated into Smoke Free (23 Limited), a leading smoking cessation app with established efficacy. The primary outcomes measured user engagement with the app.

**Methods:**

A 2-arm, parallel-group, randomized controlled trial was conducted in the United States with an 8-week follow-up. Adult individuals who smoked ≥1 cigarettes daily and planned to quit smoking within 7 days were recruited and randomized (N=500), with equal allocation. Both groups received free access to the original Smoke Free app with “core” features of its smoking cessation program (eg, a diary and craving log). The treated group received additional access to the integrated Inner Dragon game that incorporated several game mechanics designed to increase user engagement. User engagement outcomes were the number of unique app sessions, average minutes per session, days with a session, and program adherence. Self-reported and verified smoking abstinence and app satisfaction were also assessed. The main analysis estimated the intention-to-treat effect of access to Inner Dragon on each outcome. Further analyses assessed effect modification by participant characteristics and the association of intensity of game use with program adherence and abstinence.

**Results:**

Overall, user engagement was greater for treated versus control participants: they had 5.3 more sessions of Smoke Free (mean 29.6, SD 36.5 sessions vs mean 24.3, SD 37.9 sessions; *P*=.06), 0.8 more minutes per session (mean 6.9, SD 5.4 min vs mean 6.1, SD 5.2 min; *P*=.047), and 3.4 more days with a session (mean 14.3, SD 15.3 days vs mean 11.9, SD 14.3 days; *P*=.03). Program adherence, based on the number of times core features of the original Smoke Free app were used, was higher for treated versus control participants (mean 29.4, SD 41.3 times vs mean 22.6, SD 35.6 times; *P*=.03). Self-reported 7-day and 30-day point-prevalence abstinence and verified 7-day point-prevalence abstinence at 8 weeks did not significantly differ by study group. The mean repeated 1-day prevalence of quitting was higher among the treated group versus the control group (mean 17.3%, SD 25.6 vs mean 12.4%, SD 21.3; *P*=.01). App satisfaction and the motivation to (stay) quit did not differ by study group. Higher intensity of game use was associated with increased program adherence and self-reported abstinence.

**Conclusions:**

Findings suggest that the Inner Dragon game increased user engagement and program adherence. Additional refinements to the game design may clarify whether the game increases abstinence rates. Overall, it is feasible to deploy games and gamification to enhance user engagement in existing smoking cessation interventions.

**Trial Registration:**

ClinicalTrials.gov NCT05227027; https://clinicaltrials.gov/study/NCT05227027

## Introduction

### Background

Cigarette smoking remains a leading cause of preventable death and illness in the United States [1]. Although various clinic- and phone-based smoking cessation treatments are effective, the most effective cessation treatment, which combines counseling with medication, is used by fewer than 5% of individuals attempting to quit smoking [1-3]. Most individuals attempting to quit smoking do so without any assistance, resulting in success rates of <5% [4].

The advent of smartphone-delivered smoking cessation support offers a compelling alternative to traditional clinical treatments, given its potential reach and convenience. Smoking cessation apps, in particular, have gained popularity due to their low cost and just-in-time support.

Research shows that English-language smoking cessation apps have been downloaded 33 million times as of 2020 [5]. The widespread ownership of smartphones has contributed to the extensive reach of cessation apps. As of 2023, 90% of US adults, including 84% of Black adults, 91% of Hispanic adults, 79% of adults in low-income households (<US $30,000 per year), and 89% of adults aged between 50 and 64 years, owned a smartphone [6]. Consequently, smartphone-based interventions hold promise to reduce the substantial burden of tobacco-related disease and mortality.

While smoking cessation apps offer the advantage of providing timely assistance, only a small proportion of the numerous apps available in app stores have undergone rigorous testing. A systematic review conducted up until 2019 indicated that 11 smoking cessation apps had been subjected to randomized controlled trials (RCTs), although only 3 were beyond the pilot or feasibility stage [7]. Since then, there have been additional RCTs, although the literature is still emerging. Early-stage evaluations and the full-scale trials have indicated that smartphone cessation apps tend to yield intention-to-treat abstinence rates from 12% to 32% (usually at 1-2 months), with substantial variation due to differences in measures, the inclusion of bioverification, and other design factors [8-20]. However, the translation of these early-efficacy rates into real-world effectiveness remains largely uncertain, particularly regarding app features that enhance user engagement and contribute to sustained abstinence [21].

A major contributor to the modest efficacy rates for many smoking cessation apps is low user engagement and retention [22-24]. Systematic data for smartphone cessation apps are lacking, although evidence suggests a pattern of low retention. Three-quarters of mobile health apps are opened <10 times [25].

To address this gap, researchers have explored the use of serious games and gamification techniques as potential strategies to enhance user engagement and motivation [26-29]. Serious games have a primary purpose other than entertainment, such as health promotion. Gamification is a related motivational tool that uses nonmonetary rewards to make nongame activities fun or challenging. By increasing user engagement, games and gamification may increase exposure and adherence to a smoking cessation program and, in turn, increase the likelihood of quitting [30]. Games may have particular appeal to individuals who smoke, three-quarters of whom play video games, according to 1 survey [31]. Many smokers also express belief in the motivating potential of game-based approaches for smoking cessation [31,32].

Several stand-alone smartphone-based games for smoking cessation, such as Cigbreak, Crush the Crave, Inspired, QuitIT, Quittr, and Tobbstop, have been developed [12,19,20,33-39]. A few of these games have also been tested in RCTs. While users generally reported satisfaction with the gamified apps, most studies have not observed significant increases in smoking abstinence [12,19,20,38]. The variability in user engagement and retention across these apps further complicates the assessment of their overall effectiveness, with each study adopting different measurement approaches.

### This Study

We conducted an RCT to test the efficacy of a novel game intervention called Inner Dragon. Whereas prior games have been stand-alone products; Inner Dragon was integrated into the Smoke Free app (23 Limited), providing a unique opportunity to estimate the incremental benefit of embedding a game in a high-quality smoking cessation app. Smoke Free has been found to increase continuous abstinence at 6 months (12.7% intervention vs 7% comparator) on a per-protocol basis in a pragmatic RCT of 3000 participants [15]. Smoke Free is also one of the most downloaded smoking cessation apps in the Apple (Apple Inc) and Android (Google LLC) stores, with more than 800,000 downloads per year and 7 million downloads to date [14,40].

Inner Dragon is a multifaceted game iteratively developed by our team that uses virtual pet retention mechanics with some social features and customization options to promote user engagement and retention [41]. In the Inner Dragon game, users care for an evolving, customizable pet dragon, whose growth reflects the user’s own progress toward quitting smoking. Points are provided for engaging with game elements and nongame educational content. The game includes elements aimed at coping with cravings and offers asynchronous interaction with other users’ dragons. In a single-arm feasibility trial, Inner Dragon was found to have high user satisfaction and generally high user engagement [41]. This study aimed to assess whether the novel Inner Dragon game integration increased user engagement among users of a leading smartphone app for smoking cessation.

## Methods

### Trial Design

We conducted a parallel-group RCT with equal allocation to 2 groups. The treated group received free access to the Smoke Free app with the integrated Inner Dragon game. The control group received free access to the Smoke Free app without the game embedded in the app. The study protocol is provided in the supporting information with a CONSORT (Consolidated Standards of Reporting Trials) guideline checklist (Multimedia Appendix 1 [6,42-62] and Multimedia Appendix 2) [63]. The protocol was preregistered at ClinicalTrials.gov (NCT05227027). No changes to the prespecified trial design and methods were made after trial commencement. Multimedia Appendix 1 provides the study protocol.

### Recruitment and Participants

Individuals were recruited from the US-based population of general users of the Smoke Free smartphone app who downloaded the app from the Apple or Android app store. Users were invited to participate in the trial during the initial onboarding screens within the Smoke Free app via an on-screen recruitment message, “Interested in a research study to test the latest version of Smoke Free?” If a user clicked “Yes, learn more,” they were taken to a screening questionnaire to assess eligibility and a consent form in Qualtrics (Qualtrics International Inc).

Inclusion criteria were individuals aged ≥18 years living in the United States who currently smoked at least 1 cigarette per day; had downloaded the Smoke Free app; planned to quit smoking within 7 days; and were able to speak, read, and write in English. A quota of at least 100 individuals aged ≥50 years was programmed into Qualtrics to ensure the study was representative with respect to age and to enable an assessment of whether older individuals, who have a higher smoking prevalence and may be less proficient with technology or less drawn to games, had a differential response to the intervention [31,64,65]. There were no exclusion criteria.

### Randomization and Masking

Eligible, consenting participants were randomized within strata with equal allocation to one of two groups: (1) a control group that received free access to the original Smoke Free app or (2) a treated group that received free access to the Smoke Free app with the integrated Inner Dragon game. Allocation, concealed from investigators, was performed through the Qualtrics randomizer and transmitted to the Smoke Free database using the Qualtrics application programming interface. Participants were then automatically given access to the assigned intervention within the Smoke Free app. Randomization was stratified by age (<50 and ≥50 years) and smoking intensity (<5 and ≥5 cigarettes per day, on average), to facilitate the assessment of effect modification.

The assessor of the salivary cotinine test was blinded to the participants’ group assignment; other aspects of the trial were not blinded.

### Procedures

#### Overview

As part of the screening questionnaire, participants selected an initial quit date within the next 7 days. Participants were invited to use the Smoke Free app in whatever way they liked for 56 days after their planned quit date. Participants were asked via email to complete a baseline questionnaire in Qualtrics after consenting to participate in the study. The baseline questionnaire included additional questions about the person’s demographic and smoking history. A follow-up questionnaire in Qualtrics was performed 8 study weeks (56 days) after the person’s initial quit date (56-63 days after screening and consent depending on each participant’s selected quit date). An invitation to complete the follow-up questionnaire was sent by SMS text messaging. Those who reported having abstained for 7 days in the follow-up questionnaire were asked for a mailing address within the follow-up questionnaire. We then mailed them a salivary cotinine test kit and written and pictorial test instructions via 2-day mail. Following completion of the follow-up questionnaire, an initial SMS text message explained that the saliva test would be shipped shortly, and a second message sent 2 days later provided a Qualtrics link with which participants were asked to upload photos of the cotinine test results. Participants received 3 reminders via SMS text messaging and 2 via email to complete the follow-up questionnaire, and they received 3 reminders via SMS text messaging to complete the saliva testing. In-app reminders were disabled so as not to conflict with the study procedures.

#### Control Group Intervention

Participants in the control group received free access to an educational intervention consisting of the original Smoke Free app, which has previous per-protocol trial evidence of continuous abstinence at 6 months [15]. The app leverages behavior change techniques that are effective in face-to-face behavioral support programs [66]. “Core” features of the original Smoke Free app include the following: (1) a calculator that tracks the total amount of money saved by not smoking; (2) a calendar that tracks the time elapsed since the user quit smoking; (3) a scoreboard that awards badges to users for not smoking; (4) progress indicators that inform users about health improvements that the user can expect because they started their quit attempt; (5) daily missions that assign evidence-based tasks to help users avoid and resist urges to smoke (eg, noting situations when the person smokes and plans for handling those situations without smoking); (6) a diary and craving log that track the frequency, strength, and location of cravings to smoke; and (7) a text-based chatbot that delivers quitting guidance in a friendly, conversational tone. In previous randomized trials, a full version of the app with a chatbot was shown to increase user engagement compared with a reduced version of the app [67], and the daily missions were shown to increase user retention and self-reported smoking abstinence at 3 months [14]. The current core version of the Smoke Free app does not contain any additional gamification features.

#### Treated Group Intervention

Participants in the treated group received free access to core features of the original Smoke Free app as well as the integrated Inner Dragon game module. Inner Dragon uses traditional virtual pet retention with some social features and many options for customization and personalization. Virtual pets have been popular with consumers because they can foster bonding and companionship [68].

More specifically, Inner Dragon includes several game mechanics designed to increase user engagement and retention. First, the user maintains a pet dragon that hatches on the user’s quit day and evolves every 7 days to unlock new attributes and powers. The dragon acts as a virtual avatar that represents the user’s progress. Second, the user earns experience points by engaging in selected activities, including those in the game (eg, playing a minigame or feeding the dragon) and nongame features in the original Smoke Free app (eg, completing a mission or logging a diary entry). Rewards for using core app features in the original app were designed to improve adherence to the smoking cessation program. The experience points unlock gifts and cosmetics for the dragon, directly rewarding frequent and consistent use of the app. The user can customize their dragon (eg, wing shape or clothing accessories) throughout the quit experience by steadily unlocking features. Third, the Inner Dragon home screen has “care meters” that users must work on to keep from falling very low: calmness, nutrition, hygiene, and energy. Engaging with the dragon in various ways increases the readings on meters. For example, petting the dragon increases calmness, and feeding the dragon increases nutrition. Caring for and interacting with a virtual pet through the care meters can foster a bond with the pet and motivate users to return to the game regularly [68]. Fourth, the game provides tools for the user to better cope with cravings: a dragon-led breathing exercise to provide calmness and a memory minigame as a distraction. Fifth, a user can asynchronously interact with other users’ dragons in a “dragon park” by (1) viewing their profile and progress and (2) sending and receiving motivational messages and emojis from a preset menu. We hypothesize that these game mechanics increase users’ engagement with the app and, subsequently, their chance of quitting successfully.

Inner Dragon’s game design was informed by principles from the fields of psychology and behavioral economics [41]. The avatar provided salient, visual feedback with endogenous value (tied closely to the user’s motivation to quit) that may sustain and enhance motivation to quit. Self-determination theory predicts that this type of feedback is highly intrinsically motivating [69]. Furthermore, the user may identify with the digital pet as an avatar, and this may cultivate a digital therapeutic alliance with the game and app, for example, by creating a bond with the dragon and increasing the user’s confidence to succeed [42,70]. The use of frequent, salient in-game rewards was designed to counter the behavioral economic constructs of present bias and inattention to app use [71,72]. The design further included evidence-based practices from contingency management, such as the use of escalating in-game rewards for abstinence, with a reset point for lapses and sustained abstinence (by harnessing regret and loss aversion) [73]. The use of surprise gifts provided a variable reward structure designed to boost engagement and novelty [74-76]. The asynchronous interactions with other users in the dragon park provided opportunities for limited social support and social comparisons that may motivate the user to exert more effort in the quit attempt [77,78].

The various game mechanics and elements were designed to appeal to users with different motivations. For example, Yee [79] identified 3 main components of player motivation: achievement (advancement and competition), social (socializing and relationships), and immersion (discovery, customization, and escapism). The experience-point system may appeal to achievers; the interaction with other players and a sense of connection with the dragon may appeal to socializers, and the rich opportunities for dragon customization and distraction games may appeal to players seeking immersion.

A more thorough description of the design and development of Inner Dragon is described elsewhere [41].

### Measures

#### Outcome Measures

The study focused on outcomes within the domains of user engagement, abstinence from smoking, and user satisfaction and motivation.

User engagement measures included the two *primary outcome measures* of (1) the total number of unique app sessions from enrollment through 8 weeks after the user’s initial quit date, measured as the number of times the app was opened; and (2) the mean duration of app sessions, in minutes, through 8 weeks after the quit date; The *secondary outcome measures* of (3) the number of unique days with ≥1 app session, and (4) the proportion entering Inner Dragon, and the *tertiary outcome measures* of (5) program adherence, measured as the total number of times selected “core” features of the original Smoke Free app were used (reported a craving, recorded a diary entry, completed a mission, read a tip, and used the chatbot); and (6) the number of times using selected game features (opening gifts, breathing exercise, cleaning the dragon, feeding the dragon, memory minigame, using the customization menu to change appearance, and reading the dragon instruction guide). All engagement outcomes were passively collected by the app. A new session was defined as opening the app after at least 30 minutes of inactivity.

Smoking abstinence measures included the *secondary outcome measures* of (1) the proportion of participants who reported abstaining during the past 7 days at the 2-month follow-up assessment (self-reported 7-day point-prevalence abstinence), (2) the proportion of participants who reported abstaining during the past 30 days at the 2-month follow-up assessment (self-reported 30-day point-prevalence abstinence), and the *tertiary outcome measures* of (3) biochemically verified 7-day point-prevalence abstinence at the 2-month follow-up assessment, obtained from uploaded results from a self-administered salivary cotinine test (Alere iScreen Oral Fluid Device; Abbott Laboratories); and (4) repeated 1-day point-prevalence smoking abstinence, measured as the mean proportion of days each participant self-reported having abstained in the last 24 hours, collected via a pop-up box that appeared the first time each day a participant opened the app throughout the 8-week, postquit date period. Self-reported 7-day point-prevalence abstinence followed the Russell Standard, allowing for <5 slips [80]. Those who reported using nicotine replacement therapy (NRT) or electronic nicotine delivery systems (ENDSs) within the prior 7 days were coded in the main analysis as abstinent for the verified measures if no cigarettes were used [80,81].

User satisfaction and motivation measures included the *secondary outcome measures* of (1) satisfaction with the Smoke Free app (“I liked using the Smoke Free app”), reported on a 5-point Likert scale ranging from “not at all” (score=1) to “extremely” (score=5); (2) satisfaction with the Inner Dragon game (“I liked the dragon game”), reported on the same 5-point Likert scale, and the *tertiary outcome measures* of (3) rating of whether the person would recommend the assigned app to a friend on a 5-point Likert scale; (4) motivation to (stay) quit at the 8-week follow-up, reported on a 10-point scale from 0 (not at all motivated) to 10 (very motivated); and (5) digital therapeutic alliance, measured from the bonding and confidence subscales of the Mobile Agnew Relationship Measure, reported as a 16-point ordinal measure [42,70].

#### Covariates

The screening questionnaire collected demographic information on age, gender (woman, man, non-binary or other), race and ethnicity (recoded to non-Hispanic White, non-Hispanic Black, Hispanic, or other), household income category (US $0-US $19,999, US $20,000-US $39,999, US $40,000-US $59,999, US $60,000-US $79,999, US $80,000-US $99,999, and US $100,000 and more), and educational attainment (high-school diploma and less, some college or technical school, bachelor’s or associate degree, and graduate degree). Smoking characteristics at the screening included mean cigarettes per day, Fagerström Test for Nicotine Dependence (score of 0-10, where 10 is highly nicotine dependent) [82], number of past quit attempts, years since initiated, use of ENDS in last 30 days (yes or no), and use of NRT in last 30 days (yes or no). Baseline information also included frequency of video game use (not at all, less than once a month, at least monthly but not weekly, at least weekly but not every day, and every day).

### Sample Size

Power calculations were based on an anticipated sample size of 500, with an assumed 70% retention (as observed in other app-based smoking cessation trials) [12,20]. We further anticipated a mean number of app sessions of 30 (SD 45.0) per participant in the control group, based on data from past Smoke Free users provided to the study team by Smoke Free. A sample size of 500 participants with 70% retention was estimated to detect a between-group difference in app sessions, one of the primary outcomes, of 13.5 at 80% power (α=.05).

### Data Analysis

#### Main Analysis

The main analyses were conducted on an intention-to-treat basis using all randomized participants. For those outcomes measured continuously, including the 2 primary outcomes, we calculated the crude (unadjusted) difference in outcomes between each study group, using 1-tailed *t* tests of the difference in means to assess statistical significance at the 5% level. For those outcomes measured categorically, we calculated the mean difference in proportions corresponding to the risk difference and used a similar approach to assess statistical significance. For the abstinence outcomes, the intention-to-treat approach implied that those lost to follow-up were assumed to have resumed smoking (“missing=smoking”).

#### Outcome Trends

Next, we assessed visual changes over time in the number of users with a Smoke Free session, a key engagement outcome, and self-reported 1-day point-prevalence abstinence on an intention-to-treat basis (missing=smoking) and using complete cases. For each outcome, we plotted the mean for each study group by study day.

#### Intensity of Game Use

We assessed whether the intensity of game use was associated with more distal outcomes: total user engagement (total number of sessions), program adherence, and self-reported abstinence (at follow-up and repeated daily, assuming missing=smoking). This analysis, conducted among participants in the treated group only, entailed plotting estimates from local linear regressions of the number of sessions with game use on each of these outcomes, following the nonparametric approach proposed by Calonico et al [83]. The estimates included mean squared error-optimal bandwidth, 30 evaluation points. and a heteroskedasticity-robust nearest neighbor variance estimator with 3 neighbors.

#### Sensitivity and Exploratory Analyses

We examined subgroup differences across study groups for the primary engagement outcomes, in which we evaluated heterogeneity by estimating a stratified model for each covariate.

We conducted covariate-adjusted regression analyses to assess the robustness of our findings. We estimated the differences in each outcome by study group using linear regressions for continuous outcomes and logistic regressions for binary outcomes. The models adjusted for a range of prespecified potential confounders (described in the Covariates section earlier), including characteristics of participants’ sociodemographic background, smoking history, and frequency of video game use. Coefficients from the logistic regressions were expressed as risk differences, with an interpretation as a change in percentage points.

We repeated our analysis of verified abstinence—coding users of NRT or ENDS at follow-up as having not abstained. We also repeated our abstinence analysis using a complete case analysis that included only those participants who were successfully followed up. Whereas missing=smoking can lead to downward bias if loss to follow-up occurs for reasons other than relapse to smoking, complete case analysis may lead to upward bias if participants refuse to engage with follow-up because they have resumed smoking. As a further sensitivity analysis of the abstinence outcome, we estimated the treatment effect for self-reported 7-day point-prevalence abstinence from a pattern-mixture model that varied the informative missingness odds ratio between the outcome and an indicator for missingness, adjusting for covariates [84,85].

#### Adverse Events

We tabulated the number of serious adverse events by type for each study group, as reported in an open-ended text field in the follow-up questionnaire.

Analyses were conducted in Stata (version 18.0; StataCorp).

### Ethical Considerations

Ethics approval was obtained from the University of California, San Francisco institutional review board (19-29335). All participants were informed about the study details and procedures before providing their consent electronically. Participation was strictly voluntary, and participants were informed about their right to withdraw from the study at any point without any consequences. The collected data were coded and deidentified. Participants’ identities were kept confidential and stored securely in password-protected files. Coded identifiers were given to participants after obtaining informed consent. No participants’ personal identifiers were used in analyses or report writing. Participants in both groups received US $20 for completing the baseline questionnaire, US $40 for completing the follow-up questionnaire, and US $40 for completing the saliva test, if requested. All payments were provided, via email link, as a choice of a Visa (Visa Inc) prepaid card or one of hundreds of gift cards through the Tango digital payment platform (Tango Card Inc).

## Results

### Sample Characteristics

**Figure 1:**
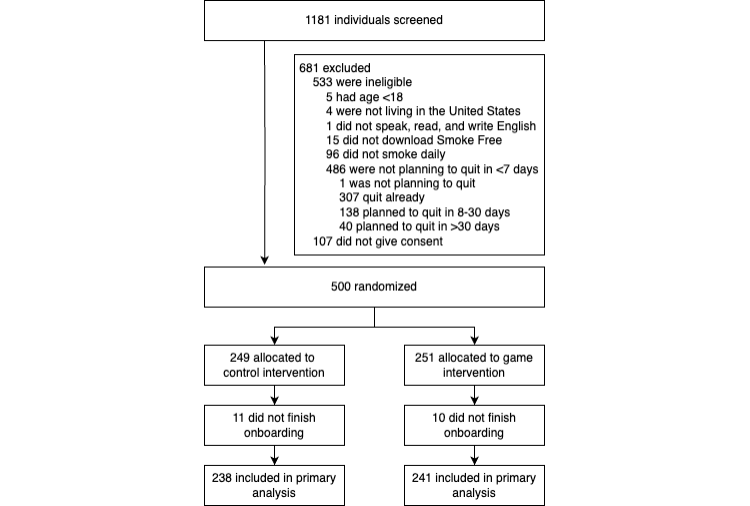
Participant flow diagram.

Figure 1 depicts the study flow diagram. We screened 1181 new Smoke Free users from February 9, 2022, through March 16, 2022, to reach our goal of enrolling 500 eligible, consenting individuals for the RCT. Of the randomized participants, 95.8% (479/500; treated: 238/251, 94.8%, and control: 241/249, 96.8%) opened Smoke Free after completing the screening questionnaire, thereby completing the onboarding process within Smoke Free. Individuals who did not finish onboarding did not return to the app after the screening survey and did not learn their allocation group. The denominator used for the primary analyses was 479.

There was a 61% (292/479) response rate for the 8-week follow-up questionnaire (treated: 154/241, 63.9% and control: 138/238, 58%), and a 71% (137/193) response rate for the saliva test (among those completing the follow-up questionnaire and reporting having quit within the prior 7 days), including 74% (73/99) of invited control participants and 68% (64/94) of invited treated participants. Nonresponse for the follow-up questionnaire and saliva test was unrelated to the study group assignment, according to *t* tests of the difference in means. However, response rates for the questionnaire and saliva test increased with the number of app sessions, and the response rate for the saliva test was higher for invited non-Hispanic Black participants (23/28, 82%) and lower for invited non-Hispanic White participants (93/136, 68.4%).

Demographic and smoking characteristics were balanced across study groups (Table 1; Table S1 in Multimedia Appendix 3 [7,8,10,12-20,86-95]). Participants were drawn from 48 states across the United States (Midwest: 126/479, 26.3%, Northeast: 90/479, 18.8%, South: 160/479, 33.4%, and West: 103/479, 21.5%). The median age of participants was 36 (IQR 29-47) years, with 19.8% (95/479) of participants aged ≥50 years due to the recruitment quota. Three-quarters (358/479, 74.7%) of participants identified as women. Most (368/479, 76.8%) were non-Hispanic White, with 10% (48/479) non-Hispanic Black or African American and 5.8% (28/479) Hispanic. Nearly 62.6% (300/479) had a household income below US $60,000, and 41.3% (198/479) had a household income <150% of the federal poverty level at the time. Most (277/479, 57.8%) did not hold a college degree. For smoking and gaming characteristics, participants were mostly moderate-to-heavy smokers (median of 15.0, IQR 10-20, cigarettes per day), had previously attempted to quit (median of 4, IQR 2-9, past attempts), and varied in terms of video game use during the prior 30 days (not at all: 116/479, 24.2% and every day: 162/479, 33.8%).

**Table 1:** Characteristics at screening of participants enrolled in the Inner Dragon trial, overall and by study group (N=479).

				Control (n=238)		Treated (n=241)		Total (N=479)
**Panel A: demographics**								
Age (y), median (IQR)				35 (28-47)		38 (29-47)		36 (29-47)
	**Gender, n (%)**							
		Women	181 (76.1)		177 (73.4)		358 (74.7)	
		Men	55 (23.1)		59 (24.5)		114 (23.8)	
		Nonbinary or other	2 (0.8)		5 (2.1)		7 (1.5)	
	**Race and ethnicity, n (%)**							
		Hispanic	9 (3.8)		19 (7.9)		28 (5.8)	
		Non-Hispanic Black	26 (10.9)		22 (9.1)		48 (10)	
		Non-Hispanic White	182 (76.5)		186 (77.2)		368 (76.8)	
		Other	21 (8.8)		14 (5.8)		35 (7.3)	
	**Household income (US $), n (%)**							
		<20,000	35 (14.7)		35 (14.5)		70 (14.6)	
		20,000-39,999	65 (27.3)		63 (26.1)		128 (26.7)	
		40,000-59,999	51 (21.4)		51 (21.2)		102 (21.3)	
		60,000-79,999	24 (10,1)		32 (13.3)		56 (11.7)	
		80,000-99,999	18 (7.6)		21 (8.7)		39 (8.1)	
		≥100,000	45 (18.9)		39 (16.2)		84 (17.5)	
	**Education, n (%)**							
		High school diploma or less	55 (23.1)		45 (18.8)		100 (20.9)	
		Some college or technical school	84 (35.3)		93 (38.6)		177 (37)	
		Bachelor’s or associate degree	68 (28.6)		83 (34.4)		151 (31.5)	
		Graduate degree	31 (13)		20 (8.3)		51 (10.6)	
**Panel B: smoking characteristics**								
	Cigarettes per day, median (IQR)		15 (10-20)		15 (10-20)		15 (10-20)	
	Fagerström Test, median (IQR)		5 (3-7)		5 (4-7)		5 (4-7)	
	Past quit attempts, median (IQR)		4 (2-8)		4 (2-9)		4 (2-9)	
	Years since initiated, median (IQR)		16 (10-28)		19 (11-29)		18 (10-29)	
	Used ENDS^a^ in last 30 days, n (%)		61 (25.6)		57 (23.7)		118 (24.6)	
	Used NRT^b^ in last 30 days, n (%)		43 (18.1)		56 (23.2)		99 (20.7)	
**Panel C: other**								
	**Frequency of playing video games, n (%)**							
		Not at all	59 (24.8)		57 (23.7)		116 (24.2)	
		Less than once a month	25 (10.5)		36 (14.9)		61 (12.7)	
		At least monthly but not weekly	25 (10.5)		26 (10.8)		51 (10.6)	
		At least weekly but not every day	40 (16.8)		49 (20.3)		89 (18.6)	
		Every day	89 (37.4)		73 (30.3)		162 (33.8)	

^a^ENDS: electronic nicotine delivery system.

^b^NRT: nicotine replacement therapy.

### Main Intervention Effects

#### User Engagement

Compared with control participants, treated participants had 5.3 more sessions of Smoke Free app use during the 8 study weeks, although this difference in the primary outcome was not statistically significant (mean 29.6, SD 36.5 sessions vs mean 24.3, SD 37.9 sessions; *P*=.06; Table 2). Treated participants used the app for more minutes per session than control participants (mean 6.9, SD 5.4 min vs 6.2, SD 5.2 min; *P*=.047 for the primary outcome) and had more days with an app session (mean 14.3, SD 15.3 days vs 13.3, SD 11.9 days; *P*=.03 for the secondary outcome). Overall, treated participants used the Inner Dragon game for an average of 7.1 (SD 14.9) days, with at least some use of each of the game features (Table S2 in Multimedia Appendix 3).

Program adherence, as measured by the number of times using certain core features in the original Smoke Free app, was greater for treated participants than control participants (mean 29.4, SD 41.3 times vs mean 22.6, SD 35.6 times; *P*=.03 for tertiary outcome). The largest increase in core features for the treated group occurred for the number of diary entries completed (mean 12.1, SD 15.9 vs mean 9.2, SD 13.2; *P*=.01 for tertiary outcome), although use of all core features increased (Table 2).

#### Smoking Abstinence

Self-reported 7-day point-prevalence abstinence on an intention-to-treat basis (assuming missing=smoking) was 39% (94/241) for the treated group and 42.4% (101/238) for the control group (difference –3.4 percentage points; *P*=.45 for secondary outcome). The difference widened when considering complete cases only, such that 61% (94/154) abstained in the treated group and 73.2% (101/138) in the control group (difference –12.1 percentage points; *P*=.03).

Verified 7-day point-prevalence abstinence at 2 months similarly showed no advantage for the treated group, such that the intention-to-treat estimates were 19.5% (47/241) in the treated group and 22.7% (54/238) in the control group (difference –3.2 percentage points; *P*=.39). This included 8.3% (20/241) of participants in the treated group and 8% (19/238) of participants in the control group who were coded abstinent because they reported having used NRT or ENDS within the prior 7 days at the 2-month follow-up. Self-reported 30-day point-prevalence abstinence at 2 months was similar among the treated group (54/241, 22.4%) and the control group (50/238, 21%) on an intention-to-treat basis (difference 1.4 percentage points; *P*=.71 for secondary outcome).

Self-reported mean repeated 1-day abstinence was higher for the treated group (mean 17.3%, SD 25.6) than for the control group (mean 12.4%, SD 21.3) on an intention-to-treat basis (difference 4.9 percentage points; *P*=.01) and among complete cases (difference 10 percentage points; *P*=.01, tertiary outcome).

#### Satisfaction and Motivation

Satisfaction with the Smoke Free app on a 5-point Likert scale (1=not at all to 5=extremely) did not differ by study group among those who completed the follow-up questionnaire (mean 3.7, SD 1.1 treated group vs mean 3.7, SD 1.0 control group; *P*=.91 for secondary outcome). Additional questions regarding satisfaction with the app were also similar between study groups (Figure S2 in Multimedia Appendix 3). Satisfaction with the Inner Dragon game (mean 3.3, SD 1.4) was a bit lower in the treated group than for the app overall.

Motivation to (stay) quit at follow-up, measured on a 10-point scale (0=not at all motivated to 10=very motivated), did not differ significantly (mean 9.3, SD 1.3 treated group vs mean 9.3, SD 1.3 control group; *P*=.87 for tertiary outcome); however, a Qualtrics programming error led to missing data for 51% (148/290) of respondents. The digital therapeutic alliance also did not differ by the study group at follow-up (mean 12.7, SD 2.2 treated group vs mean 12.9, SD 2.4 control group; *P*=.75).

**Table 2:** Comparison of means for user engagement, smoking abstinence, and user satisfaction according to study group assignment.

				Control		Treated		Difference in means		*P* value^a^		Observations, n^b^
**Panel A: user engagement**												
	App sessions^c^, mean (SD)			24.3 (37.9)		29.6 (36.5)		5.3		.06		479
	Minutes per session^c^, mean (SD)			6.1 (5.2)		6.9 (5.4)		0.9		.047		479
	Days with session, mean (SD)			11.9 (13.3)		14.3 (15.3)		2.5		.03		479
	Game sessions, mean (SD)			—^d^		7.1 (14.9)		—		—		241
	Index of core feature use, mean (SD)			22.6 (35.6)		29.4 (41.3)		6.8		.03		479
	**Use of core features, mean (SD)**											
		Cravings reported	1.9 (4.1)		2.7 (5.0)		0.8		.02		479	
		Diary entries	9.2 (13.2)		12.1 (15.9)		3.0		.01		479	
		Missions completed	3.8 (8.8)		4.9 (10.2)		1.0		.12		479	
		Chatbot sessions	4.0 (9.9)		5.4 (11.5)		1.4		.08		479	
	Index of game feature use, mean (SD)			—		112.9 (258.6)		—		—		241
**Panel B: point**-**prevalence abstinence**												
	**7-day abstinence at 2 months, % (n/N)**											
		Self-reported, missing=smoking	42.4 (101/238)		39 (94/241)		–3.4		.45		479	
		Self-reported, complete cases	73.2 (101/138)		61 (94/154)		–12.1		.03		292	
		Verified^e^, missing=smoking	22.7 (54/238)		19.5 (47/241)		–3.2		.39		479	
		Verified^e^, complete cases	74 (54/73)		73.4 (47/64)		–0.5		.94		137	
	**30-day abstinence at 2 months, % (n/N)**											
		Self-reported, missing=smoking	21 (50/238)		22.4 (54/241)		1.4		.71		479	
		Self-reported, complete cases	36.2 (50/138)		35.1 (54/154)		–1.2		.84		292	
	**Mean repeated 1-day abstinence, mean (SD)**											
		Self-reported, missing=smoking	12.4 (21.3)		17.3 (25.6)		4.9		.01		479	
		Self-reported, complete cases	55 (42.8)		65 (39)		10.0		.01		401	
**Panel C: satisfaction and motivation**												
	Satisfaction with app^f^, mean (SD)			3.7 (1.1)		3.7 (1.0)		0		.91		271
	Satisfaction with game^g^, mean (SD)			—		3.3 (1.4)		—		—		84
	Recommend app to friends^h^, mean (SD)			4.0 (1.1)		3.8 (1.1)		–0.2		.20		271
	Motivation to (stay) quit, mean (SD)			9.4 (1.5)		9.3 (1.3)		0		.87		144
	Digital therapeutic alliance, mean (SD)			12.9 (2.4)		12.7 (2.2)		–0.2		.75		268

^a^The *P* value is derived from a *t* test of the crude (unadjusted) difference in means between the treated and control groups.

^b^The number of observations (rightmost column) varies by outcome based on whether it was collected passively or in the follow-up survey, as well as whether it applies to all participants or treated participants only.

^c^Denotes a primary outcome measure.

^d^Not applicable. Applies to the treated group only.

^e^Codes as abstinent those who self-reported abstinence but reported use of nicotine replacement therapy or vaping products within the last 7 days.

^f^“I liked using the Smoke Free app” on a Likert scale ranging from “not at all” (1) to “extremely” (5).

^g^“I liked Inner Dragon” on a Likert scale ranging from “not at all” (1) to “extremely” (5).

^h^“I would recommend the Smoke Free App to a friend who wants to quit” on a Likert scale ranging from “not at all” (1) to “extremely” (5).

### Outcome Trends

**Figure 2:**
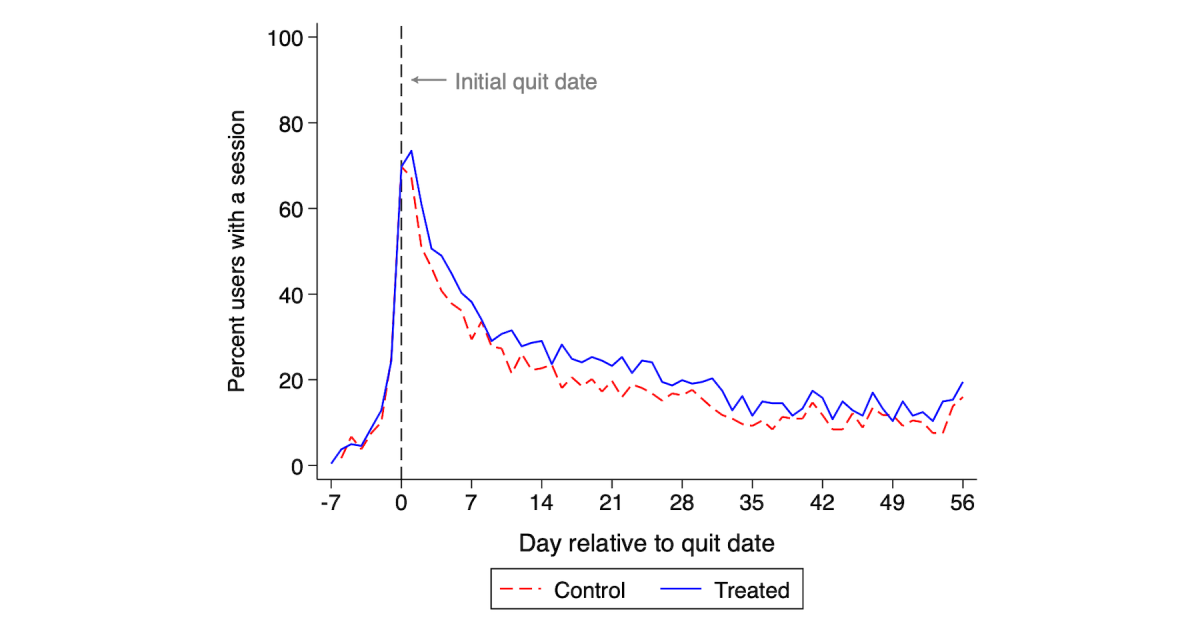
Percentage of participants with any sessions of Smoke Free app use by day in the game intervention (treated) group versus the control group (N=479).

Days with any app sessions decreased for both study groups over time, from roughly 80% on the person’s quit day to roughly 20% on day 56 (Figure 2). The treated group retained slightly increased use throughout this period.

**Figure 3:**
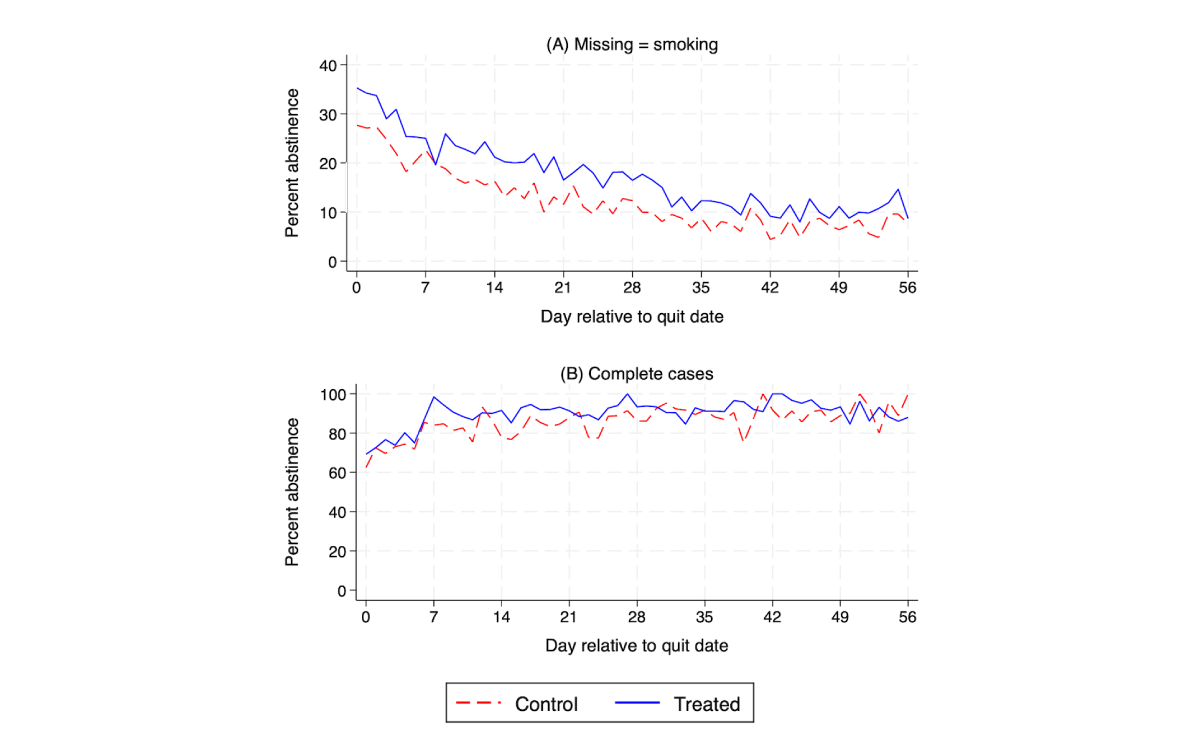
Percentage of participants with self-reported repeated 1-day point-prevalence abstinence by day in the game intervention (treated) group versus control group. (A) This panel assumes that missing reports are current smokers. (B) This panel uses complete cases only.

While the share of participants who reported having abstained the prior day decreased for both study groups, the treated group retained its advantage over the control group throughout the study period when analyzing the data on an intention-to-treat basis (Figure 3A). When analyzing complete cases only, we observe that the percentage of participants who reported having abstained remained high throughout the study period, and the magnitude of the difference between study groups was not as great as when assuming missing=smoking (Figure 3B).

### Intensity of Game Use

**Figure 4:**
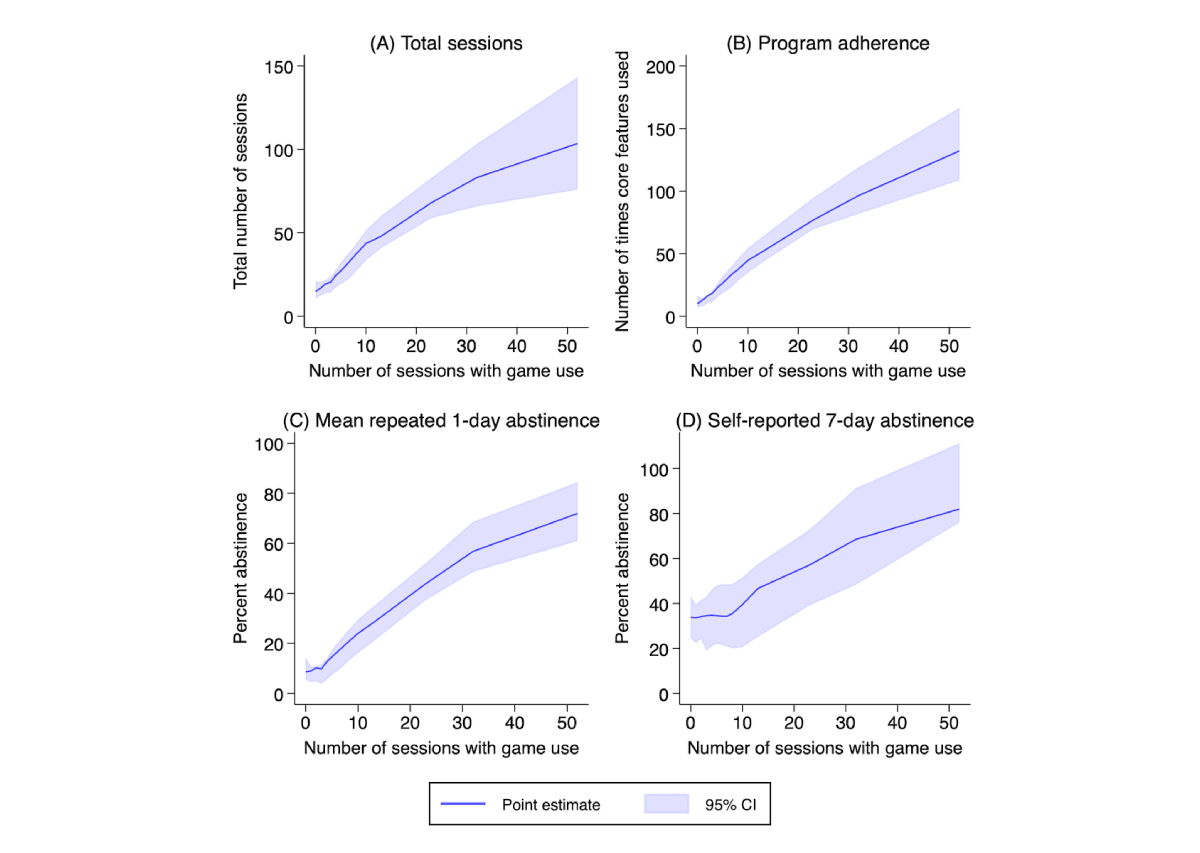
Association of intensity of game use with selected outcomes. The shaded area denotes the 95% CI.

Treated participants who had more sessions of game use had more app sessions overall, which is a mechanical relationship. More sessions of game use was also associated with increased program adherence (Figure 4B), increased mean repeated 1-day abstinence (Figure 4C), and 7-day abstinence at follow-up (Figure 4D). A 1 SD in sessions of game use (from 0-15 game sessions) was associated with a 4-fold increase in program adherence and an increase in self-reported 7-day point-prevalence abstinence at follow-up of 12.9 percentage points (from 33.8%, 95% CI 24.5%-43.2%, to 46.7%, 95% CI 25.2%-57.5%).

### Sensitivity Analyses

#### Subgroup Analyses

The effects of Inner Dragon on user engagement did not significantly vary with participant characteristics, according to subgroup analyses, although certain patterns emerged (Figure S6 in Multimedia Appendix 3). In unadjusted analyses, the treatment effect on the total number of app sessions was greater for younger participants (aged <50 years) and non-Hispanic White participants compared with non-Hispanic Black or Hispanic participants and decreased with household income. Furthermore, the effects were greater for those with low nicotine dependence, fewer (<4) past quit attempts, and use of e-cigarettes in the last 30 days. Intervention effects did not vary by video gaming history.

#### Covariate-Adjusted Analyses

Adjusting for participant characteristics (Table 1) as covariates, the differences in user engagement outcomes by study group were similar to, but slightly smaller in magnitude than, the crude differences for number of app sessions (coefficient 4.6, 95% CI –2.2 to 11.3; *P*=.18), average minutes per session (coefficient 0.8, 95% CI –0.2 to 1.9; *P*=.11), days with a session (coefficient 2.2, 95% CI –0.4 to 4.7; *P*=.10), and program adherence (coefficient 6.6, 95% CI –0.4 to 13.6; *P*=.06; Table S3 in Multimedia Appendix 3). The effect sizes for self-reported and verified 7-day and self-reported 30-day point-prevalence abstinence remained similar to the crude differences in magnitude. Estimates, on an intention-to-treat basis, were –2.4 percentage points (95% CI –11.1 to 6.4; *P*=.60) for self-reported 7-day abstinence, –2.7 percentage points (95% CI –10.0 to 4.6; *P*=.47) for verified 7-day abstinence, and 1.4 percentage points (95% CI –6.0 to 8.9; *P*=.70) for self-reported 30-day abstinence. Mean repeated 1-day abstinence remained higher for the treated group than the control group (difference 4.5 percentage points, 95% CI 0.2 to 8.7; *P*=.04) on an intention-to-treat basis (Table S3 in Multimedia Appendix 3).

#### Sensitivity of Abstinence Estimates

Coding NRT and ENDS users as not abstinent, verified 7-day point-prevalence estimates were similar to the main results (difference 4.3 percentage points, 95% CI –1.8 to 10.5; *P*=.16). Results from the pattern-mixture model indicated that the treatment effect estimates for abstinence were highly sensitive to assumptions about the nature of the missing outcomes (Figure S1 in Multimedia Appendix 3). Assuming missingness is random (equivalent to missing=smoking), there would be no difference in abstinence between groups, whereas assuming informative missingness, there would be reduced abstinence in the treated group compared with the control group.

### Adverse Events

There were no serious adverse events reported during the trial. In total, 1.2% (3/241) reported adverse events in the treated group and 2.9% (7/239) in the control group. Adverse events were feelings of withdrawal, craving, or irritability (1 report in the treated group and 4 in the control group); being anxious or depressed (1 report in the treated group and 3 in the control group); and constipation (1 report in the treated group and 0 in the control group).

## Discussion

### Principal Findings

Our RCT assessed the efficacy of the novel Inner Dragon game module integrated into the Smoke Free app for promoting user engagement. The study of 479 participants revealed that treated individuals exhibited some increased user engagement metrics, including a (nonsignificant) 21.8% increase in the number of app sessions and a significant 20.2% increase in days of app use. Engaging with Inner Dragon led users in the treated group to use core features of the original Smoke Free app significantly (30.1%) more often, and this increase in smoking cessation program adherence implies that game use complemented (“crowded in”) rather than replaced (“crowded out”) use of the nongame content. User engagement is a fundamental concern for mobile health apps [22-24], and our findings support the role of gamification as a key driver of user engagement.

Smoking abstinence outcomes showed a nuanced picture, with self-reported 7-day point-prevalence abstinence favoring the control group and repeated 1-day abstinence favoring the treated group. In exploratory analyses, we found that this difference was driven in part by treated participants with missing point-prevalence abstinence data having much higher mean repeated abstinence than control participants with missing point-prevalence abstinence data (mean 54.5% SD 5.5 vs mean 35.4% SD 4.9, difference 19.1 percentage points; *P*=.01). Nevertheless, improving user engagement and program adherence metrics did not definitively produce higher smoking abstinence rates in this trial. One possibility is that gamifying the quit process may have undermined participants’ focus on the higher purpose of quitting smoking. Further research into features of the game, user characteristics, and the original app may be warranted to understand the relationship between user engagement and smoking abstinence.

Finally, the study found a positive association between the intensity of game use and both program adherence and smoking abstinence, suggesting that game-driven engagement may support downstream outcomes.

### Comparison With Previous Studies

In the context of the existing literature on smartphone apps for smoking cessation, our findings align with studies emphasizing the importance of user engagement in digital interventions [22-24]. Notably, the increased engagement observed in our study was associated with positive outcomes such as greater program adherence in the form of higher core feature use. To our knowledge, the link between user engagement and program adherence has not been explored with regard to games for smoking cessation, in part because previously evaluated games have been stand-alone products [96].

We found some evidence of effect modification, such that user engagement was greater for younger and lower-income users. Our results echo the call for personalized interventions, consistent with research suggesting the impact of individual characteristics on engagement and outcomes in digital health interventions [97]. Furthermore, our exploration of gamification elements in the Inner Dragon game aligns with literature highlighting the potential of gaming features to enhance engagement and promote behavior change in smoking cessation apps [96]. Prior studies of smartphone-based games for smoking cessation have found mixed evidence regarding smoking abstinence [19,20,38,98], although study quality in the broader literature has generally been deemed fair or poor with important methodological limitations [98]. We found that the bundled game intervention as a whole contributed to the effects of Inner Dragon on increased user engagement. We were not able to isolate the effect of any individual feature of Inner Dragon; exploratory analyses revealed that participants’ use of most key game features was moderately or strongly positively associated with the total number of app sessions (range: 0.39-0.74), and each game feature was strongly or very strongly positively associated with other game features (range: 0.53-1.00; Figure S5 in Multimedia Appendix 3). A future study might use a factorial design to identify the most important game features for stimulating user engagement and abstinence.

Our study supports the integration of personalized strategies, such as games in digital interventions, tailored to individual characteristics and broadly appealing to users with different motivations (eg, achievement, socializing, and immersion), to optimize user engagement and intervention effectiveness. Emerging capabilities of artificial intelligence may facilitate this process [99]. Relatedly, self-paced initiatives such as gamified smoking cessation apps constitute an alternative to traditional smoking cessation interventions that often depend on advanced scheduling. Many researchers have sought ways to leverage technological advancements that enable the delivery of just-in-time, tailored support for behavior change, although large-scale studies on the effectiveness of smoking cessation are lacking [100].

Comparing our abstinence rates to previous studies of smoking cessation apps is difficult due to important differences in study design, such as intervention delivery, comparator, follow-up period, abstinence measure, and the use of bioverification. Focusing on 14 RCTs with follow-up of 1 to 3 months, abstinence ranged widely from 1.6% (234/14,228) to 81.8% (9/11) in the treated groups and 0.9% (124/13,884) to 66.2% (190/287) in the control groups, and intention-to-treat intervention effects ranged from –0.9 to 11.5 percentage points (Table S4 in Multimedia Appendix 3). We observed abstinence within these ranges, although abstinence in our control group was relatively high (101/238, 42.4%), suggesting that the original Smoke Free app was a particularly effective comparator group on its own. Our study is also distinguished from other app-based studies for having collected high-frequency (daily) abstinence data as surveillance within a smoking cessation intervention; however, there is, related literature on just-in-time adaptive interventions that collect high-frequency data with the aim of identifying smoking triggers and delivering real-time feedback [100].

### Strengths and Limitations

This study has important strengths. First, this study is one of the largest randomized trials of a smartphone-based intervention involving games or gamification for smoking cessation. Second, to our knowledge, it is the first study to assess a game that is integrated into an existing smoking cessation app. By integrating Inner Dragon into one of the most downloaded smoking cessation apps, which also has established effectiveness, we were able to evaluate the added benefit of the game module for user engagement. The game intervention can be readily scaled to the 800,000 users who download Smoke Free each year; Inner Dragon is planned to become available to general users of Smoke Free starting in 2024. Third, passive collection of use data ensured that our primary outcome data were complete, accurate, and able to be tracked longitudinally. Fourth, the availability of high-frequency, longitudinal abstinence data was unique for studies of smoking cessation games and uncommon for studies of smoking cessation interventions more generally [101].

However, this study has several limitations. First, the short-term follow-up limits our ability to assess sustained abstinence outcomes. Second, the generalizability of our findings may be constrained by the demographic composition of the app-recruited study population. It is noteworthy that our study drew from a general population of users of the Smoke Free app, a diverse user base in a real-world context. Yet, the use of the Smoke Free app may not be representative of all smoking cessation apps, and the rapidly evolving landscape of digital interventions may influence the generalizability of our findings over time. Third, our game intervention includes a bundle of interlocking features, and we cannot disentangle their individual effects. Fourth, while our study was adequately powered for our primary outcomes, we may have been underpowered for some secondary outcomes, such as smoking abstinence, as well as for subgroup analyses.

### Conclusions

In conclusion, our study contributes valuable insights to the growing body of literature on digital smoking cessation interventions involving serious games, emphasizing the role of user engagement. The positive associations between game use with program adherence and smoking cessation outcomes underscore the potential of gamification interventions in promoting behavior change. Future research should extend follow-up durations, refine personalized interventions, evaluate effectiveness in a real-world setting, and address methodological challenges, such as missing data, to guide the development of evidence-based digital health interventions for smoking cessation.

## Appendix

Multimedia Appendix 1 Trial protocol.

Multimedia Appendix 2 CONSORT (Consolidated Standards of Reporting Trials) checklist.

Multimedia Appendix 3 Supplemental tables and figures.

## Abbreviations

CONSORT: Consolidated Standards of Reporting Trials
ENDS: electronic nicotine delivery system
NRT: nicotine replacement therapy
RCT: randomized controlled trial

## Data Availability

The survey datasets generated and analyzed during this study are publicly available [102]. The app use data generated and analyzed during this study are not publicly available due to a third-party agreement between the University of California San Francisco and 23 Limited (the developer of Smoke Free) that prohibits data sharing.

## References

[1] US Department of Health and Human Services (2014). The Health Consequences of Smoking—50 Years of Progress: A Report of the Surgeon General.

[2] Babb S, Malarcher A, Schauer G, Asman K, Jamal A (2017). Quitting smoking among adults — United States, 2000–2015. MMWR Morb Mortal Wkly Rep.

[3] U.S. National Cancer Institute and World Health Organization (2016). The economics of tobacco and tobacco control. National Cancer Institute.

[4] Rafful C, García-Rodríguez O, Wang S, Secades-Villa R, Martínez-Ortega JM, Blanco C (2013). Predictors of quit attempts and successful quit attempts in a nationally representative sample of smokers. Addict Behav.

[5] Abroms LC, Lee Westmaas J, Bontemps-Jones J, Ramani R, Mellerson J (2013). A content analysis of popular smartphone apps for smoking cessation. Am J Prev Med.

[6] (2024). Mobile fact sheet. Pew Research Center.

[7] Chu KH, Matheny SJ, Escobar-Viera CG, Wessel C, Notier AE, Davis EM (2021). Smartphone health apps for tobacco cessation: a systematic review. Addict Behav.

[8] Bricker JB, Mull KE, Kientz JA, Vilardaga R, Mercer LD, Akioka KJ, Heffner JL (2014). Randomized, controlled pilot trial of a smartphone app for smoking cessation using acceptance and commitment therapy. Drug Alcohol Dependence.

[9] Iacoviello BM, Steinerman JR, Klein DB, Silver TL, Berger AG, Luo SX, Schork NJ (2017). Clickotine, a personalized smartphone app for smoking cessation: initial evaluation. JMIR Mhealth Uhealth.

[10] BinDhim NF, McGeechan K, Trevena L (2018). Smartphone smoking cessation application (SSC App) trial: a multicountry double-blind automated randomised controlled trial of a smoking cessation decision-aid 'app'. BMJ Open.

[11] Marler JD, Fujii CA, Utley DS, Tesfamariam LJ, Galanko JA, Patrick H (2019). Initial assessment of a comprehensive digital smoking cessation program that incorporates a mobile app, breath sensor, and coaching: cohort study. JMIR Mhealth Uhealth.

[12] Krebs P, Burkhalter J, Fiske J, Snow H, Schofield E, Iocolano M, Borderud S, Ostroff JS (2019). The QuitIT coping skills game for promoting tobacco cessation among smokers diagnosed with cancer: pilot randomized controlled trial. JMIR Mhealth Uhealth.

[13] Bricker JB, Watson NL, Mull KE, Sullivan BM, Heffner JL (2020). Efficacy of smartphone applications for smoking cessation: a randomized clinical trial. JAMA Intern Med.

[14] Crane D, Ubhi HK, Brown J, West R (2018). Relative effectiveness of a full versus reduced version of the 'smoke free' mobile application for smoking cessation: an exploratory randomised controlled trial. F1000Res.

[15] Jackson SE, Kale D, Beard E, Perski O, West R, Brown J Effectiveness of the offer of the smoke free smartphone application compared with no intervention for smoking cessation: a pragmatic randomised controlled trial. medRxiv. Preprint posted online on January 12, 2023.

[16] Krishnan N, Elf JL, Chon S, Golub JE (2019). COach2Quit: a pilot randomized controlled trial of a personal carbon monoxide monitor for smoking cessation. Nicotine Tob Res.

[17] Goldenhersch E, Thrul J, Ungaretti J, Rosencovich N, Waitman C, Ceberio MR (2020). Virtual reality smartphone-based intervention for smoking cessation: pilot randomized controlled trial on initial clinical efficacy and adherence. J Med Internet Res.

[18] Herbec A, Brown J, Shahab L, West R, Raupach T (2019). Pragmatic randomised trial of a smartphone app (NRT2Quit) to improve effectiveness of nicotine replacement therapy in a quit attempt by improving medication adherence: results of a prematurely terminated study. Trials.

[19] Pallejà-Millán M, Rey-Reñones C, Barrera Uriarte ML, Granado-Font E, Basora J, Flores-Mateo G, Duch J (2020). Evaluation of the Tobbstop mobile app for smoking cessation: cluster randomized controlled clinical trial. JMIR Mhealth Uhealth.

[20] Baskerville NB, Struik LL, Guindon GE, Norman CD, Whittaker R, Burns C, Hammond D, Dash D, Brown KS (2018). Effect of a mobile phone intervention on quitting smoking in a young adult population of smokers: randomized controlled trial. JMIR Mhealth Uhealth.

[21] Paige SR, Alber JM, Stellefson ML, Krieger JL (2018). Missing the mark for patient engagement: mHealth literacy strategies and behavior change processes in smoking cessation apps. Patient Educ Couns.

[22] Amagai S, Pila S, Kaat AJ, Nowinski CJ, Gershon RC (2022). Challenges in participant engagement and retention using mobile health apps: literature review. J Med Internet Res.

[23] Meyerowitz-Katz G, Ravi S, Arnolda L, Feng X, Maberly G, Astell-Burt T (2020). Rates of attrition and dropout in app-based interventions for chronic disease: systematic review and meta-analysis. J Med Internet Res.

[24] Baumel A, Muench F, Edan S, Kane JM (2019). Objective user engagement with mental health apps: systematic search and panel-based usage analysis. J Med Internet Res.

[25] Bowman D (2011). Motivating patients to use smartphone health apps. Fierce Healthcare.

[26] Cafazzo JA, Casselman M, Hamming N, Katzman DK, Palmert MR (2012). Design of an mHealth app for the self-management of adolescent type 1 diabetes: a pilot study. J Med Internet Res.

[27] Primack BA, Carroll MV, McNamara M, Klem ML, King B, Rich M, Chan CW, Nayak S (2012). Role of video games in improving health-related outcomes: a systematic review. Am J Prev Med.

[28] King D, Greaves F, Exeter C, Darzi A (2013). 'Gamification': influencing health behaviours with games. J R Soc Med.

[29] Looyestyn J, Kernot J, Boshoff K, Ryan J, Edney S, Maher C (2017). Does gamification increase engagement with online programs? A systematic review. PLoS One.

[30] Richardson A, Graham AL, Cobb N, Xiao H, Mushro A, Abrams D, Vallone D (2013). Engagement promotes abstinence in a web-based cessation intervention: cohort study. J Med Internet Res.

[31] Raiff BR, Jarvis BP, Rapoza D (2012). Prevalence of video game use, cigarette smoking, and acceptability of a video game-based smoking cessation intervention among online adults. Nicotine Tob Res.

[32] Oliver JA, Hallyburton MB, Pacek LR, Mitchell JT, Vilardaga R, Fuemmeler BF, McClernon FJ (2018). What do smokers want in a smartphone-based cessation application?. Nicotine Tob Res.

[33] Edwards EA, Caton H, Lumsden J, Rivas C, Steed L, Pirunsarn Y, Jumbe S, Newby C, Shenvi A, Mazumdar S, Smith JQ, Greenhill D, Griffiths CJ, Walton RT (2018). Creating a theoretically grounded, gamified health app: lessons from developing the Cigbreak smoking cessation mobile phone game. JMIR Serious Games.

[34] Baskerville NB, Struik LL, Dash D (2018). Crush the crave: development and formative evaluation of a smartphone app for smoking cessation. JMIR Mhealth Uhealth.

[35] Raiff BR, Fortugno N, Scherlis DR, Rapoza D (2018). A mobile game to support smoking cessation: prototype assessment. JMIR Serious Games.

[36] Krebs P, Burkhalter JE, Snow B, Fiske J, Ostroff JS (2013). Development and alpha testing of QuitIT: an interactive video game to enhance skills for coping with smoking urges. JMIR Res Protoc.

[37] Bindoff I, de Salas K, Peterson G, Ling T, Lewis I, Wells L, Gee P, Ferguson SG (2016). Quittr: the design of a video game to support smoking cessation. JMIR Serious Games.

[38] Bindoff I, Ling TR, Gee P, Geelan B, Ferguson SG, Peterson GM (2020). Effects of a mobile app called Quittr, which utilizes premium currency and games features, on improving engagement with smoking cessation intervention: pilot randomized controlled trial. JMIR Serious Games.

[39] Marin-Gomez FX, Garcia-Moreno Marchán R, Mayos-Fernandez A, Flores-Mateo G, Granado-Font E, Barrera Uriarte ML, Duch J, Rey-Reñones C (2019). Exploring efficacy of a serious game (Tobbstop) for smoking cessation during pregnancy: randomized controlled trial. JMIR Serious Games.

[40] Jackson SE, Perski O, Crane D, Michie S, West R, Brown J (2019). Effectiveness of an offer of the smoke free smartphone application for smoking cessation: protocol for a randomized controlled trial. Addiction.

[41] White JS, Salem MK, Toussaert S, Westmaas JL, Raiff BR, Crane D, Warrender E, Lyles C, Abroms L, Thrul J (2023). Developing a game (inner dragon) within a leading smartphone app for smoking cessation: design and feasibility evaluation study. JMIR Serious Games.

[42] D'Alfonso S, Lederman R, Bucci S, Berry K (2020). The digital therapeutic alliance and human-computer interaction. JMIR Ment Health.

[43] Henley SJ, Thomas CC, Sharapova SR, Momin B, Massetti GM, Winn DM, Armour BS, Richardson LC (2016). Vital signs: disparities in tobacco-related cancer incidence and mortality - United States, 2004-2013. MMWR Morb Mortal Wkly Rep.

[44] Irvin Vidrine J, Reitzel LR, Wetter DW (2009). The role of tobacco in cancer health disparities. Curr Oncol Rep.

[45] Fiscella K, Franks P, Doescher MP, Saver BG (2002). Disparities in health care by race, ethnicity, and language among the insured: findings from a national sample. Med Care.

[46] Hargraves JL, Hadley J (2003). The contribution of insurance coverage and community resources to reducing racial/ethnic disparities in access to care. Health Serv Res.

[47] Cokkinides VE, Halpern MT, Barbeau EM, Ward E, Thun MJ (2008). Racial and ethnic disparities in smoking-cessation interventions: analysis of the 2005 national health interview survey. Am J Prev Med.

[48] Franks P, Fiscella K, Meldrum S (2005). Racial disparities in the content of primary care office visits. J Gen Intern Med.

[49] Ubhi HK, Michie S, Kotz D, Wong WC, West R (2015). A mobile app to aid smoking cessation: preliminary evaluation of SmokeFree28. J Med Internet Res.

[50] Bricker JB, Copeland W, Mull KE, Zeng EY, Watson NL, Akioka KJ, Heffner JL (2017). Single-arm trial of the second version of an acceptance and commitment therapy smartphone application for smoking cessation. Drug Alcohol Depend.

[51] van den Brand FA, Nagelhout GE, Winkens B, Chavannes NH, van Schayck OC (2018). Effect of a workplace-based group training programme combined with financial incentives on smoking cessation: a cluster-randomised controlled trial. Lancet Public Health.

[52] Augustson E, Cole-Lewis H, Sanders A, Schwarz M, Geng Y, Coa K, Hunt Y (2017). Analysing user-reported data for enhancement of SmokefreeTXT: a national text message smoking cessation intervention. Tob Control.

[53] Theng YL, Lee JW, Patinadan PV, Foo SS (2015). The use of videogames, gamification, and virtual environments in the self-management of diabetes: a systematic review of evidence. Games Health J.

[54] Patel MS, Benjamin EJ, Volpp KG, Fox CS, Small DS, Massaro JM, Lee JJ, Hilbert V, Valentino M, Taylor DH, Manders ES, Mutalik K, Zhu J, Wang W, Murabito JM (2017). Effect of a game-based intervention designed to enhance social incentives to increase physical activity among families: the BE FIT randomized clinical trial. JAMA Intern Med.

[55] Roepke AM, Jaffee SR, Riffle OM, McGonigal J, Broome R, Maxwell B (2015). Randomized controlled trial of SuperBetter, a smartphone-based/internet-based self-help tool to reduce depressive symptoms. Games Health J.

[56] El-Hilly AA, Iqbal SS, Ahmed M, Sherwani Y, Muntasir M, Siddiqui S, Al-Fagih Z, Usmani O, Eisingerich AB (2016). Game on? Smoking cessation through the gamification of mHealth: a longitudinal qualitative study. JMIR Serious Games.

[57] Hall SM, Delucchi KL, Velicer WF, Kahler CW, Ranger-Moore J, Hedeker D, Tsoh JY, Niaura R (2001). Statistical analysis of randomized trials in tobacco treatment: longitudinal designs with dichotomous outcome. Nicotine Tob Res.

[58] Hedeker D, Mermelstein RJ, Demirtas H (2007). Analysis of binary outcomes with missing data: missing = smoking, last observation carried forward, and a little multiple imputation. Addiction.

[59] Nelson DB, Partin MR, Fu SS, Joseph AM, An LC (2009). Why assigning ongoing tobacco use is not necessarily a conservative approach to handling missing tobacco cessation outcomes. Nicotine Tob Res.

[60] Blankers M, Smit ES, van der Pol P, de Vries H, Hoving C, van Laar M (2016). The missing=smoking assumption: a fallacy in internet-based smoking cessation trials?. Nicotine Tob Res.

[61] Raghunathan TE, Lepkowski JM, Van Hoewyk J, Solenberger P (2001). A multivariate technique for multiply imputing missing values using a sequence of regression models. Survey Methodol.

[62] van Buuren S (2007). Multiple imputation of discrete and continuous data by fully conditional specification. Stat Methods Med Res.

[63] Eldridge SM, Chan CL, Campbell MJ, Bond CM, Hopewell S, Thabane L, Lancaster GA (2016). CONSORT 2010 statement: extension to randomised pilot and feasibility trials. BMJ.

[64] Upton CR, Nastasi JA, Raiff BR (2022). Identifying video game preferences among adults interested in quitting smoking cigarettes: survey study. JMIR Serious Games.

[65] Cornelius ME, Loretan CG, Jamal A, Davis Lynn BC, Mayer M, Alcantara IC, Neff L (2023). Tobacco product use among adults – United States, 2021. MMWR Morb Mortal Wkly Rep.

[66] Ubhi HK, Michie S, Kotz D, van Schayck OC, Selladurai A, West R (2015). Characterising smoking cessation smartphone applications in terms of behaviour change techniques, engagement and ease-of-use features. Behav Med Pract Policy Res.

[67] Perski O, Crane D, Beard E, Brown J (2019). Does the addition of a supportive chatbot promote user engagement with a smoking cessation app? An experimental study. Digit Health.

[68] Bylieva D, Almazova N, Lobatyuk V, Rubtsova A (2019). Virtual pet: trends of development. Proceedings of the 2019 International Conference on Digital Science.

[69] Ryan RM, Deci EL (2000). Self-determination theory and the facilitation of intrinsic motivation, social development, and well-being. Am Psychol.

[70] Berry K, Salter A, Morris R, James S, Bucci S (2018). Assessing therapeutic alliance in the context of mHealth interventions for mental health problems: development of the mobile Agnew relationship measure (mARM) questionnaire. J Med Internet Res.

[71] Chaloupka FJ, Levy MR, White JS (2019). Estimating biases in smoking cessation: evidence from a field experiment (article no. 26522). National Bureau of Economic Research.

[72] Goldin J, Homonoff T (2013). Smoke gets in your eyes: cigarette tax salience and regressivity. Am Econ J Econ Policy.

[73] Roll JM, Higgins ST (2000). A within-subject comparison of three different schedules of reinforcement of drug abstinence using cigarette smoking as an exemplar. Drug Alcohol Depend.

[74] Shen L, Fishbach A, Hsee CK (2015). The motivating-uncertainty effect: uncertainty increases resource investment in the process of reward pursuit. J Consum Res.

[75] Golman R, Loewenstein G, Molnar A, Saccardo S (2022). The demand for, and avoidance of, information. Manag Sci.

[76] Buechel EC, Li R (2023). Mysterious consumption: preference for horizontal (vs. vertical) uncertainty and the role of surprise. J Consum Res.

[77] Meeker D, Linder JA, Fox CR, Friedberg MW, Persell SD, Goldstein NJ, Knight TK, Hay JW, Doctor JN (2016). Effect of behavioral interventions on inappropriate antibiotic prescribing among primary care practices: a randomized clinical trial. JAMA.

[78] Allcott H, Rogers T (2014). The short-run and long-run effects of behavioral interventions: experimental evidence from energy conservation. Am Econ Rev.

[79] Yee N (2006). Motivations for play in online games. Cyberpsychol Behav.

[80] West R, Hajek P, Stead L, Stapleton J (2005). Outcome criteria in smoking cessation trials: proposal for a common standard. Addiction.

[81] Benowitz NL, Bernert JT, Foulds J, Hecht SS, Jacob P, Jarvis MJ, Joseph A, Oncken C, Piper ME (2020). Biochemical verification of tobacco use and abstinence: 2019 update. Nicotine Tob Res.

[82] Heatherton TF, Kozlowski LT, Frecker RC, Fagerström KO (1991). The Fagerström test for nicotine dependence: a revision of the Fagerström Tolerance Questionnaire. Br J Addict.

[83] Calonico S, Cattaneo MD, Farrell MH (2018). On the effect of bias estimation on coverage accuracy in nonparametric inference. J Am Stat Assoc.

[84] White IR, Horton NJ, Carpenter J, Pocock SJ (2011). Strategy for intention to treat analysis in randomised trials with missing outcome data. BMJ.

[85] White IR, Carpenter J, Horton NJ (2018). A mean score method for sensitivity analysis to departures from the missing at random assumption in randomised trials. Stat Sin.

[86] Hertzberg JS, Carpenter VL, Kirby AC, Calhoun PS, Moore SD, Dennis MF, Dennis PA, Dedert EA, Beckham JC (2013). Mobile contingency management as an adjunctive smoking cessation treatment for smokers with posttraumatic stress disorder. Nicotine Tob Res.

[87] Masaki K, Tateno H, Nomura A, Muto T, Suzuki S, Satake K, Hida E, Fukunaga K (2020). A randomized controlled trial of a smoking cessation smartphone application with a carbon monoxide checker. NPJ Digit Med.

[88] Webb J, Peerbux S, Smittenaar P, Siddiqui S, Sherwani Y, Ahmed M, MacRae H, Puri H, Bhalla S, Majeed A (2020). Preliminary outcomes of a digital therapeutic intervention for smoking cessation in adult smokers: randomized controlled trial. JMIR Ment Health.

[89] Whittaker R, McRobbie H, Bullen C, Rodgers A, Gu Y, Dobson R (2019). Mobile phone text messaging and app-based interventions for smoking cessation. Cochrane Database Syst Rev.

[90] Barnett A, Ding H, Hay KE, Yang IA, Bowman RV, Fong KM, Marshall HM (2020). The effectiveness of smartphone applications to aid smoking cessation: a meta-analysis. Clin eHealth.

[91] Cobos-Campos R, de Lafuente AS, Apiñaniz A, Parraza N, Llanos IP, Orive G (2020). Effectiveness of mobile applications to quit smoking: systematic review and meta-analysis. Tob Prev Cessat.

[92] Haskins BL, Lesperance D, Gibbons P, Boudreaux ED (2017). A systematic review of smartphone applications for smoking cessation. Transl Behav Med.

[93] Regmi K, Kassim N, Ahmad N, Tuah NA (2017). Effectiveness of mobile apps for smoking cessation: a review. Tob Prev Cessat.

[94] Staiger PK, O'Donnell R, Liknaitzky P, Bush R, Milward J (2020). Mobile apps to reduce tobacco, alcohol, and illicit drug use: systematic review of the first decade. J Med Internet Res.

[95] Ybarra ML, Jiang Y, Free C, Abroms LC, Whittaker R (2016). Participant-level meta-analysis of mobile phone-based interventions for smoking cessation across different countries. Prev Med.

[96] Derksen ME, van Strijp S, Kunst AE, Daams JG, Jaspers MW, Fransen MP (2020). Serious games for smoking prevention and cessation: a systematic review of game elements and game effects. J Am Med Inform Assoc.

[97] Perski O, Blandford A, West R, Michie S (2017). Conceptualising engagement with digital behaviour change interventions: a systematic review using principles from critical interpretive synthesis. Transl Behav Med.

[98] Rajani NB, Bustamante L, Weth D, Romo L, Mastellos N, Filippidis FT (2023). Engagement with gamification elements in a smoking cessation app and short-term smoking abstinence: quantitative assessment. JMIR Serious Games.

[99] Hassoon A, Baig Y, Naiman DQ, Celentano DD, Lansey D, Stearns V, Coresh J, Schrack J, Martin SS, Yeh HC, Zeilberger H, Appel LJ (2021). Randomized trial of two artificial intelligence coaching interventions to increase physical activity in cancer survivors. NPJ Digit Med.

[100] Perski O, Hébert ET, Naughton F, Hekler EB, Brown J, Businelle MS (2022). Technology-mediated just-in-time adaptive interventions (JITAIs) to reduce harmful substance use: a systematic review. Addiction.

[101] Businelle MS, Ma P, Kendzor DE, Frank SG, Wetter DW, Vidrine DJ (2016). Using intensive longitudinal data collected via mobile phone to detect imminent lapse in smokers undergoing a scheduled quit attempt. J Med Internet Res.

[102] (2024). Evaluating the impact of a game (Inner Dragon) on user engagement within a leading smartphone app for smoking cessation: a randomized controlled trial. OSF Home.

