# Supplementary material for: Evaluating the Impact of a Game (Inner Dragon) on User Engagement Within a Leading Smartphone App for Smoking Cessation: Randomized Controlled Trial
Source: J Med Internet Res. 2024 Oct 30;26:e57839. doi: 10.2196/57839 (PMC11561441; doi:10.2196/57839)
Supplement: Multimedia Appendix 1 [file jmir_v26i1e57839_app1.pdf]

Influence of a Game Embedded in a Smartphone App for Smoking Cessation on User Engagement: A Randomized Pilot Trial

National Clinical Trial (NCT) Identified Number: NCT05227027

Principal Investigator: Justin S. White, PhD
Sponsor: University of California, San Francisco

Funded by: National Cancer Institute (R21 CA238301), National Institute of Aging (P30 AG012839), the Center on the Economics and Demography of Aging at the University of California, Berkeley, and the Hellman Fellows Fund

Approved by: University of California San Francisco Institutional Review Board (IRB # 19-29335)

Version Number: v1.0

4 February 2022

CONFIDENTIALITY STATEMENT

This document is confidential communication. Acceptance of this document constitutes agreement by the recipient that no unpublished information contained herein will be published or disclosed without prior approval of the Principal Investigator or other participating study leadership and as consistent with the NIH terms of award.

Table of Contents

STATEMENT OF COMPLIANCE 1
INVESTIGATOR'S SIGNATURE 2
1 PROTOCOL SUMMARY 3
1.1 Synopsis 3
1.2 Schema 4
1.3 Schedule of Activities 4
2 KEY ROLES 5
3 INTRODUCTION 6
3.1 Study Rationale 6
3.2 Background 6
3.3 risk/benefit assessment 8
3.3.1 Known Potential Risks 8
3.3.2 Known Potential Benefits 8
3.3.3 Assessment of Potential Risks and Benefits 8
4 OBJECTIVES AND ENDPOINTS 9
5 STUDY DESIGN 12
5.1 Overall Design 12
5.2 Scientific Rationale for Study Design 12
5.3 Justification for Intervention 13
5.4 End-of-Study Definition 13
6 STUDY POPULATION 13
6.1 Inclusion Criteria 13
6.2 Exclusion Criteria 14
6.3 Lifestyle Considerations 14
6.4 Screen Failures 14
6.5 Strategies for Recruitment and Retention 14
7 STUDY INTERVENTION 14
7.1 Study Intervention 14
7.1.1 Study Intervention Description 14
7.1.2 Administration and/or Dosing 15
7.2 Fidelity 15
7.2.1 Interventionist Training and Tracking 15
7.3 Measures to Minimize Bias: Randomization and Blinding 15
7.4 Study Intervention/Experimental Manipulation Adherence 16
7.5 Concomitant Therapy 16
7.5.1 Rescue Therapy 16
8 STUDY INTERVENTION/EXPERIMENTAL MANIPULATION DISCONTINUATION AND PARTICIPANT DISCONTINUATION/WITHDRAWAL 16
8.1 Discontinuation of Study Intervention/Experimental Manipulation 16
8.2 Participant Discontinuation/Withdrawal from the Study 16
8.3 Lost to Follow-Up 17
9 STUDY ASSESSMENTS AND PROCEDURES 17
9.1 Endpoint and Other Non-Safety Assessments 17
9.2 Safety Assessments 18
9.3 Adverse Events and Serious Adverse Events 18
9.3.1 Definition of Adverse Events 18

9.3.2 Definition of Serious Adverse Events ... 18
9.3.3 Classification of an Adverse Event ... 18
9.3.4 Time Period and Frequency for Event Assessment and Follow-Up ... 19
9.3.5 Adverse Event Reporting ... 19
9.3.6 Serious Adverse Event Reporting ... 19
9.3.7 Reporting Events to Participants ... 20
9.3.8 Events of Special Interest ... 20
9.3.9 Reporting of Pregnancy ... 20
9.4 Unanticipated Problems ... 20
9.4.1 Definition of Unanticipated Problems ... 20
9.4.2 Unanticipated Problems Reporting ... 20
9.4.3 Reporting Unanticipated Problems to Participants ... 21
10 STATISTICAL CONSIDERATIONS ... 21
10.1 Statistical Hypotheses ... 21
10.2 Sample Size Determination ... 21
10.3 Populations for Analyses ... 22
10.4 Statistical Analyses ... 22
10.4.1 General Approach ... 22
10.4.2 Analysis of the Primary Endpoint(s) ... 22
10.4.3 Analysis of the Secondary Endpoint(s) ... 23
10.4.4 Safety Analyses ... 23
10.4.5 Baseline Descriptive Statistics ... 23
10.4.6 Planned Interim Analyses ... 23
10.4.7 Sub-Group Analyses ... 23
10.4.8 Tabulation of Individual Participant Data ... 23
10.4.9 Exploratory Analyses ... 24
11 SUPPORTING DOCUMENTATION AND OPERATIONAL CONSIDERATIONS ... 24
11.1 Regulatory, Ethical, and Study Oversight Considerations ... 24
11.1.1 Informed Consent Process ... 24
11.1.2 Study Discontinuation and Closure ... 24
11.1.3 Confidentiality and Privacy ... 25
11.1.4 Future Use of Stored Specimens and Data ... 25
11.1.5 Key Roles and Study Governance ... 25
11.1.6 Safety Oversight ... 26
11.1.7 Clinical Monitoring ... 26
11.1.8 Quality Assurance and Quality Control ... 26
11.1.9 Data Handling and Record Keeping ... 26
11.1.10 Protocol Deviations ... 27
11.1.11 Publication and Data Sharing Policy ... 28
11.1.12 Conflict of Interest Policy ... 28
11.2 Additional Considerations ... 29
11.3 Abbreviations and Special Terms ... 29
11.4 Protocol Amendment History ... 31
12 REFERENCES ... 32

STATEMENT OF COMPLIANCE

This trial will be carried out in accordance with International Council on Harmonisation Good Clinical Practice (ICH GCP) and the following:

- United States (US) Code of Federal Regulations (CFR) applicable to clinical studies (45 CFR Part 46, 21 CFR Part 50, 21 CFR Part 56, 21 CFR Part 312, and/or 21 CFR Part 812).

National Institutes of Health (NIH)-funded investigators and clinical trial site staff who are responsible for the conduct, management, or oversight of NIH-funded clinical trials have completed Human Subjects Protection Training.

The protocol, informed consent form(s), recruitment materials, and all participant materials will be submitted to the IRB for review and approval. Approval of both the protocol and the consent form(s) must be obtained before any participant is consented. Any amendment to the protocol will require review and approval by the IRB before the changes are implemented to the study. All changes to the consent form(s) will be IRB approved; a determination will be made regarding whether a new consent needs to be obtained from participants who provided consent, using a previously approved consent form.

INVESTIGATOR'S SIGNATURE

The signature below constitutes the approval of this protocol and provides the necessary assurances that this study will be conducted according to all stipulations of the protocol, including all statements regarding confidentiality, and according to local legal and regulatory requirements and applicable US federal regulations and ICH guidelines, as described in the *Statement of Compliance* above.

Principal Investigator or Clinical Site Investigator:

Signed: ____ Date: ____________________

Name: Justin S. White, PhD

Title: Associate Professor

Investigator Contact Information
Affiliation*: University of California, San Francisco
Address: 490 Illinois Street, Box 0936, San Francisco, CA 94158
Telephone: 415-476-8045
Email: justin.white@ucsf.edu

1 PROTOCOL SUMMARY

1.1 SYNOPSIS

**Title:**	Influence of a Game Embedded in a Smartphone App for Smoking Cessation on User Engagement: A Randomized Pilot Trial
**Study Description:**	In this study, we test the effects of a video game embedded in a commercially available mobile app for smoking cessation. Our hypothesis is that the game increases engagement, retention, and smoking cessation rates compared with a version of the app without the game. We will test this hypothesis in a 500-person pilot trial, with one-half of participants randomized to receive the core app plus embedded game, and one-half of participants randomized to receive the core app only.
**Objective:**	Our objective is to test the study feasibility, engagement, retention, and early efficacy of a 'gamified' smoking cessation app, compared to one without the game.
**Endpoints:**	Primary Endpoints: Engagement objectively measured by 1) total number of unique app sessions and 2) time spent per session.<br><br>Secondary Endpoints: Early efficacy measured by 7- and 30-day point prevalence abstinence, engagement measured by days of use, satisfaction measured by rating of the app and game.
**Study Population:**	500 new adult users of the Smoke Free app who smoked 1 or more cigarettes daily prior to download will be enrolled in this study. We will set a quota to include ≥100 individuals age ≥50 years old to explore differential program effects by age.
**Phase:**	2/3
**Description of Sites/Facilities Enrolling Participants:**	Participants will be enrolled via recruitment banners during the onboarding screens in the Smoke Free app. The study will take place in the Smoke Free app.
**Description of Study Intervention:**	The study intervention is a video game in which users i) raise a pet dragon that develops in tandem with the quit attempt and ii) earn points for using game and non-game features within the app.
**Study Duration:**	Approximately 4 months from when the study opens for enrollment until completion of data collection
**Participant Duration:**	Approximately 9 weeks for each participant to complete all tasks

1.2 SCHEMA

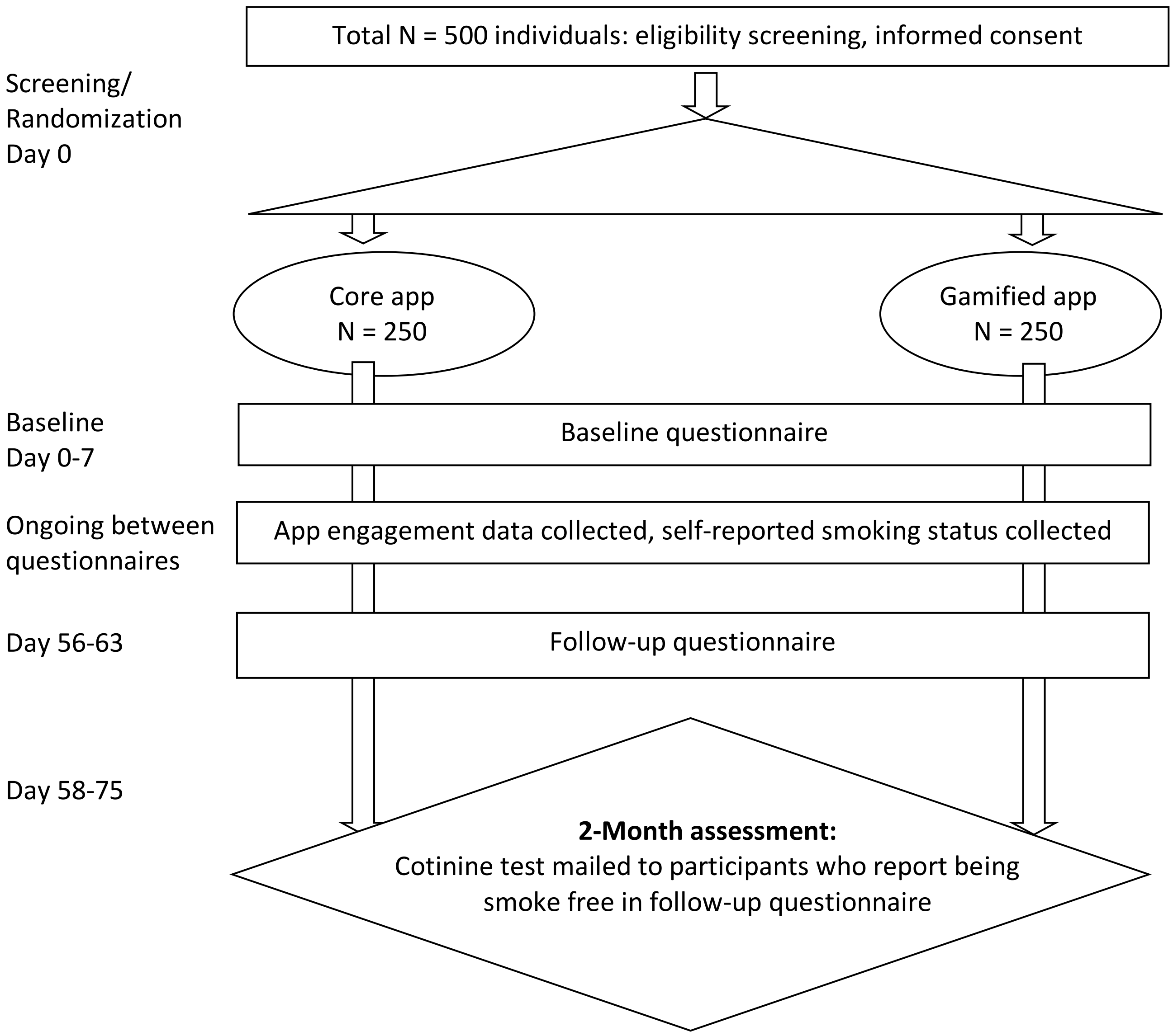

1.3 SCHEDULE OF ACTIVITIES

Please see schematic of study design above.

Individuals will complete the screening questionnaire when they first open the Smoke Free app (Day 0). Informed consent occurs at the end of the screening questionnaire. Randomization occurs immediately following the screening questionnaire within the Smoke Free app. A link to the baseline questionnaire will be sent on Day 0. Participants have until Day 7 to complete the baseline questionnaire. The person's quit date will occur on Day 0-7 (within 7 days of enrollment). Objective data will be passively collected in the app from enrollment until 8 weeks after the person's initial quit date (Day 56-63). Eight weeks post-

quit date (in Day 56-63), the person will receive an email link to the follow-up questionnaire. If the person reports having abstained from smoking for 7 or more days, they will be mailed the same or next business day a saliva cotinine test kit sent by 2-day mail (arriving Day 58-68). The participant will complete the saliva test within 7 days. Thus, the 2-month assessment will be completed between Day 58-75.

2 KEY ROLES

**Principal Investigator:**	Justin S. White, PhD<br>University of California, San Francisco<br>490 Illinois Street, Box 0936<br>San Francisco, CA 94158<br>tel: 1-415-476-8045<br>justin.white@ucsf.edu
**Study Coordinator:**	Marie Salem<br>University of California, San Francisco<br>490 Illinois Street, Box 0936<br>San Francisco, CA 94158<br>marie.salem@ucsf.edu
**Other Key Personnel:**	Lorien C. Abroms, ScD<br>George Washington University
	Courtney R. Lyles, PhD<br>University of California, San Francisco
	Bethany R. Raiff, PhD<br>Rowan University
	Johannes Thrul, PhD<br>Johns Hopkins University
	Severine Toussaert, PhD<br>University of Oxford
	J. Lee Westmaas, PhD<br>American Cancer Society
**Software Developers:**	David Crane, PhD<br>23 Limited
	Ed Warrender<br>23 Limited

3 INTRODUCTION

3.1 STUDY RATIONALE

Enhancing the effectiveness of tobacco cessation interventions would significantly reduce premature deaths from lung cancer and other tobacco-related diseases. Smartphone apps for smoking cessation are one promising approach, as more smokers turn to their smartphones for assistance with quitting. However, these programs tend to have high drop-out rates. "Gamification" is a motivational tool designed to increase engagement in mHealth apps. It employs non-monetary rewards to transform non-game contexts into fun or a challenge. Yet, gamification has rarely been tested for smoking cessation apps, despite its potential to increase engagement. In this study, we will test the effects of gamification in a mobile smoking cessation setting.

To do this, we will conduct a randomized pilot trial that compares the effectiveness of a popular smoking cessation app called Smoke Free with and without a video game module. Our goal will be to determine whether the game module increases engagement, retention, and smoking abstinence compared with an app with core features only.

3.2 BACKGROUND

Tobacco use is the leading preventable cause of death and disease in the US.[1] While multiple clinic- and phone-based smoking cessation treatments have been proven effective, most smoking cessation attempts are unaided and successful less than 5% of the time.[2] The most effective cessation treatment—counseling plus medication—is used by fewer than 5% of those attempting to quit.[1, 3, 4] Racial/ethnic and socioeconomic disparities in tobacco-related cancer incidence and mortality are persistent and well documented,[5, 6] driven in part by minority and low-income groups typically having less access to health care[7, 8] and being offered clinical treatments for smoking cessation less frequently than their counterparts.[9, 10]

Smartphone cessation apps present an expanding opportunity for reducing the population disease burden of tobacco use given their potential reach and growing popularity. The population-level impact of tobacco cessation interventions is driven by the number of smokers they reach and their effectiveness. Our research shows that Android cessation apps are downloaded up to 1.2 million times per month.[11] The low cost and convenience of smartphone-delivered support makes them an appealing alternative to clinical treatments for many smokers. The enormous reach of cessation apps has been aided by growing smartphone ownership. As of 2018, 77% of US adults owned a smartphone, including three-quarters of black and Hispanic adults, two-thirds of adults in low-income households (<$30,000 per year), and 73% of adults age 50-64.[12] Thus, smartphone-based interventions hold great promise to reduce the high burden and disparities in tobacco-related cancer risk and mortality.

Smartphone cessation apps are especially well suited to delivering timely assistance. Yet few of the hundreds of apps available in app stores have been tested rigorously—only 3 in RCTs,

according to a review through 2015.[13] Early-stage evaluations, including subsequent ones, have found that smartphone cessation apps generate 1- to 2-month abstinence rates ranging from 21-29%.[14-21] It is as yet unclear how these efficacy rates will translate into real-world effectiveness.

A major contributor to the modest efficacy rates for many real-world mHealth cessation interventions is low retention. In a study of >25,000 users of NCI's SmokefreeTXT, among the largest text messaging cessation programs nationwide, 61% dropped out before the 6-week program ended, due in large part to failed quit attempts.[22] Systematic data for smartphone cessation apps is lacking, although evidence suggests a similar pattern of low retention. Three-quarters of mHealth apps are opened <10 times.[23] In one evaluation of a cessation app (N = 1,669), >70% used the app <7 days.[15] This is consistent with studies of other cessation apps that have found program completion rates of 24% and 40%.[16, 20] It is critically important to find effective ways to engage users in smartphone interventions, to increase program retention and efficacy.

Our study aims to identify approaches to motivate, engage, and retain smokers in cessation programs, which is critically important for curbing the burden of tobacco-related disease. Behavioral researchers agree that video games and gamification may be potent tools for health behavior change.[24-26] Games are designed to increase users' motivation and engagement, which in turn increases exposure and adherence to the tobacco cessation program (Figure 1).[27] In our game design, users maintain a digital pet that evolves as the user's quit attempt progresses. This provides a visual feedback mechanism that has "endogenous" value (tied closely to users' motivation to quit) that may sustain and enhance motivation to quit. The game also may increase motivation through the limited social support and opportunities for social comparisons afforded by the park component in the game. The game also provides tools for the user to better cope with the challenges of withdrawal: a mini-game as a distraction and a breathing exercise to provide calm. Further, the user may identify with the digital pet as an avatar, and this may cultivate a digital therapeutic alliance with the game and app, e.g., by creating a bond with the dragon and increasing the user's confidence to succeed.[28, 29] All of these pathways—engagement, retention, program adherence, motivation, coping, and digital therapeutic alliance—are hypothesized to combine to increase the user's chance of quitting successfully.

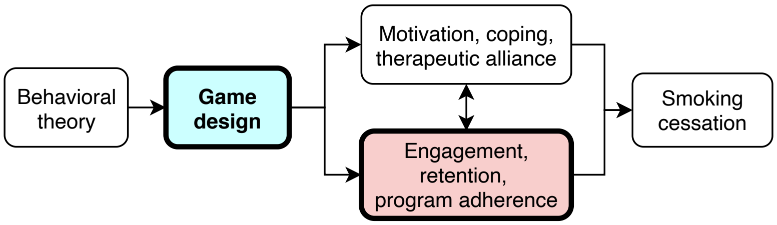

Our primary outcomes relate to the relationship between our game design and user engagement in the program. A recent systematic review of 15 trials and >10,000 participants concluded that gamification promoted engagement in online programs, though only one of those trials focused on health.[30] Other studies show that gamification can promote physical activity, diabetes self-management, and mental health, supporting the scientific premise of the proposed study; however, these have typically had small samples and non-randomized designs.[31-33] Early-stage work has also shown that games and gamification in cessation apps are

feasible and acceptable to smokers,[34, 35] Several cessation apps have included gamification elements.[20] Yet, little is known about the effects of video games and gamification for smoking cessation, despite the potential it has shown in other health domains. We seek to fill this critical knowledge gap by generating preliminary data toward demonstrating the practical benefits of a theory-informed game intervention for reducing tobacco use.

3.3 RISK/BENEFIT ASSESSMENT

3.3.1 KNOWN POTENTIAL RISKS

This study poses a minimal risk to participants. Study participation is completely voluntary, and participants may discontinue participation at any time without prejudice. Regarding study questionnaires, participants may experience psychological discomfort in discussing personal health information or negative experiences with tobacco use. As with any study, there is the potential for loss of confidentiality. A remote possibility is that users may substitute their nicotine dependence for a dependence on the game. The game is designed for brief, limited interactions and is unlikely to provide sufficient reinforcement to be a platform for addiction.

Appropriate precautions will be taken to mitigate risks, such as loss of confidentiality. These include the use of unique study codes for participants and password-protected computers for data storage. Compliance with all Institutional Review Board (IRB) regulations concerning data collection, data storage, and data destruction will be strictly observed. Data will only be accessible to research personnel and will be stored and coded according to guidelines set forth by the overseeing IRB.

3.3.2 KNOWN POTENTIAL BENEFITS

Participants may learn about reducing tobacco use. This may increase their ability to quit smoking during the study or during future quit attempts. Participants may have a feeling of contributing to knowledge in the health and social sciences. The research presents a reasonable opportunity to further the understanding and prevention of serious tobacco-related health conditions.

3.3.3 ASSESSMENT OF POTENTIAL RISKS AND BENEFITS

The study poses minimal risks to participants, but offers an opportunity to improve understanding of smoking cessation and to enhance smoking cessation rates, which can have large health benefits for the participant and any family members who are exposed to second-hand smoke. We believe that these benefits outweigh any risks.

4 OBJECTIVES AND ENDPOINTS

OBJECTIVES	ENDPOINTS	JUSTIFICATION FOR ENDPOINTS
Primary		
Our primary objective is to test the engagement of users of a 'gamified' smoking cessation smartphone app, compared to one without the game.	<u>Primary Endpoints:</u><br>Engagement:<br>1. Number of unique app sessions: number of times the app was opened; passively collected by the app and averaged in analysis to calculate mean number of app opens by study group over the study period. A new session is defined as use of the app after at least 30 minutes of inactivity.<br>2. Minutes of app usage per session: passively collected by the app and averaged in analysis among those with a session to calculate mean number of minutes of usage per session by study group.	The game intervention is designed to increase the engagement of users, either in terms of the number of times the user interacts with the app or the time spent with the app each time the person opens the app. These endpoints have been used in prior trials of smartphone-based cessation interventions.[36] Increased engagement in the quit attempt, in turn, is expected to be associated with increases in program retention, program adherence, and smoking abstinence.
Secondary		
Our secondary objective is to test alternative measures of user engagement and more distal outcomes of a 'gamified' smoking cessation app on user behavior, namely retention and early efficacy, compared with one without the game.	<u>Secondary Endpoints:</u><br>1. Engagement: number of unique days with at least one app session.<br>2. Early efficacy:<br>a. Self-reported 7-day point-prevalence smoking abstinence at 2 months post-quit date; proportion of participants who report having abstained for the prior 7 days during the 2-month follow-up assessment. Recoded to 0 if the person reported having smoked during the last 7 days during repeated measurement of daily smoking in the app.<br>b. Self-reported 30-day point-prevalence smoking abstinence at 2 months post-quit date [early efficacy]: Proportion of participants who report having abstained for the prior 30 days during the 2-month follow-up assessment. Recoded to 0 if the	• Early efficacy, as measured by short-term smoking abstinence, is assumed to mediate the relationship between user engagement with the app and long-term smoking abstinence.<br>• User satisfaction is a measure of the acceptability of the intervention for users.

OBJECTIVES	ENDPOINTS	JUSTIFICATION FOR ENDPOINTS
	person reported having smoked over the last 7 days during repeated measurement of daily smoking in the app.<br>3. Satisfaction:<br>a. Rating of satisfaction with assigned app on a 5-point Likert scale from "Not at all" (1) to "Extremely" (5), assessed in the 2-month follow-up questionnaire. The measure is dichotomized at a threshold of "Somewhat" (3) or higher. Averaged by study group.<br>b. Rating of satisfaction with game (intervention group only) on a 5-point Likert scale from "Not at all" (1) to "Extremely" (5), assessed in the 2-month follow-up questionnaire. The measure is dichotomized at a threshold of "Somewhat" (3) or higher. Averaged by study group.	
Tertiary/Exploratory		
Our tertiary objective is to test further measures of key outcomes of a 'gamified' smoking cessation app, compared with one with core app features only. These include repeated point-prevalence smoking abstinence, adherence to core app features, engagement with various game features, satisfaction, motivation to quit, and digital therapeutic alliance.	Tertiary Endpoints:<br>4. Early efficacy:<br>a. Biochemically verified 7-day point-prevalence smoking abstinence at 2 months post-quit date; proportion of participants who report having abstained for the prior 7 days during the 2-month follow-up assessment, verified by saliva cotinine test. Averaged by study group. Recoded to 0 if the person reported having smoked during the same 7-day window during repeated measurement of daily smoking in the app.<br>b. Biochemically verified 30-day point-prevalence smoking abstinence at 2 months post-quit date [early efficacy]: Proportion of participants who report having abstained for the prior 30 days during the 2-month follow-up assessment, verified by saliva cotinine test. Averaged by study group. Recoded to 0 if the person reported having smoked over the same 30-day window	

OBJECTIVES	ENDPOINTS	JUSTIFICATION FOR ENDPOINTS
	during repeated measurement of daily smoking in the app.<br>c. Repeated 1-day point-prevalence smoking abstinence; proportion of participants who self-report having abstained in the last 24 hours, collected via a pop-up box that appears the first time each day a participant opens the app.<br>5. Program adherence: Number of non-game (core app) activities completed in the app by activity type: passively collected by the app to assess number of times the participant completed the following activity types: reported a craving, recorded a diary, completed a mission, read a tip, used the chatbot.<br>6. Engagement:<br>a. Number of in-game gifts unlocked during the study period (intervention group only); a summary measure of intensity of engagement (dose of treatment) of the app; passively collected by the app.<br>b. Number of game activities completed in the app by activity type; passively collected by the app to assess number of times the participant completed the following activity types: navigated to the game dashboard, completed each pet care activity (breathing exercise, feeding, washing, mini-game), visited the park, sent a message to another user in the park, used the customization menu to change appearance.<br>7. Satisfaction: Rating of whether would recommend assigned app to a friend on a 5-point Likert scale from “Not at all” (1) to “Extremely” (5), assessed in the 2-month follow-up questionnaire. The measure is dichotomized at a threshold of “Somewhat” (3) or higher.<br>8. Motivation to quit: motivation to quit, reported on a 10-point scale from not at all motivated (0) to very unmotivated (10). Measured in follow-up questionnaire.	

OBJECTIVES	ENDPOINTS	JUSTIFICATION FOR ENDPOINTS
	9. Digital therapeutic alliance: bonding and confidence sub-scales, reported on a 4-point scale. The measure is dichotomized at a threshold of "Agree" (3) or higher. Measured in follow-up questionnaire.	

5 STUDY DESIGN

5.1 OVERALL DESIGN

The pilot trial will be a two-arm randomized trial of 500 adult users of the Smoke Free app. We will set a quota to include at least 100 individuals age ≥ 50 years old to explore differential program effects by age. The goal of the pilot trial is to assess whether a video game increased engagement with the app, relative to a version of the app without the game. The primary outcome is the number of app opens per participant.

Our primary hypothesis is that the app with the game will increase a user's engagement, as objectively measured by number of unique app sessions and average minutes of app usage per session. Our secondary hypotheses are that the app with the game will increase retention, early efficacy, and program adherence (use of core app features).

During the study period, participants will be randomized in a 1:1 ratio to the core version of the Smoke Free app plus the game module or to the same core version of the Smoke Free app without the game module (a two-arm trial). Assessors of the follow-up cotinine test to assess smoking status will be blinded; other research personnel will not be. Individuals will be invited to participate in the pilot trial in the initial onboarding screens within the Smoke Free smartphone app, with a link to a screening questionnaire and consent form in Qualtrics. We will recruit individuals age ≥ 18 years old living in the US who smoke at least 1 cigarette per day and have downloaded the Smoke Free app, plan to quit smoking within 7 days, are able to speak, read, and write in English, and are willing to provide a saliva test sample. Participation will last until 8 weeks (56 days) after the person's selected quit day.

Participants will be asked to complete a baseline questionnaire in Qualtrics after consenting to participate in the study and a follow-up questionnaire in Qualtrics at the end of the 56-day period. Those reporting being smoke-free in the follow-up questionnaire will be mailed a salivary cotinine test kit to upload photos of the test results that biochemically verify smoking status.

The main engagement analyses will be conducted on an intent-to-treat basis using all randomized participants. Regression models will adjust for a range of potential confounders, including gender, age, race/ethnicity, cigarettes per day at baseline, and years smoked.

5.2 SCIENTIFIC RATIONALE FOR STUDY DESIGN

The study design is a two-arm, pilot randomized controlled trial because the objective of the study is to test the feasibility, acceptability, and short-term proof of concept of the intervention. The trial design enables us to assess the incremental benefit of including elements of the game in a high-quality smoking cessation app.

5.3 JUSTIFICATION FOR INTERVENTION

Smartphone apps have become an increasingly popular mode by which people who smoke try to quit, yet the currently available evidence indicates that retention in smartphone-based smoking cessation programs is low. It is, therefore, important to test interventions that promote user engagement and retention in smartphone-based smoking cessation programs. Gamification has been incorporated into many apps under the belief that it promotes user engagement, although there is limited evidence on games and gamification in smartphone apps for smoking cessation. Our game intervention was designed using principles from psychology and behavioral economics to promote user engagement within an existing smoking cessation app.

The intervention accommodates users who have different levels of interest in the game, including those who wish to have brief, occasional interactions and those who seek more intensive forms of engagement with the app.

5.4 END-OF-STUDY DEFINITION

A participant is considered to have completed the study if they have remained in the study for 56 days since their selected quit date, completed the follow-up assessment, and if applicable completed and returned results from the saliva cotinine test.

6 STUDY POPULATION

6.1 INCLUSION CRITERIA

To be eligible to participate in this study, an individual must meet all of the following criteria:

1. Provides electronic informed consent.
2. Male or female, aged 18 and older
3. Has downloaded and opened the Smoke Free app
4. A smoker of at least 1 cigarette per day
5. Plans to quit smoking within the next 7 days
6. Speaks, reads, and writes English
7. Lives in the United States

6.2 EXCLUSION CRITERIA

None

6.3 LIFESTYLE CONSIDERATIONS

N/A

6.4 SCREEN FAILURES

Screen failures are defined as participants who consent to participate in this study but are not subsequently assigned to the study intervention or entered in the study. Screen failures will be able to use the Smoke Free app without restriction (dependent on the level of membership that they decide to purchase). Screen failures might occur in the event of a programming error in Qualtrics survey, an error in the Qualtrics API, or the Smoke Free software code.

6.5 STRATEGIES FOR RECRUITMENT AND RETENTION

Pilot trial participants will be recruited from among the population of general users of the Smoke Free smartphone app. Users will be invited to participate in the pilot trial during the initial on-boarding screens within the Smoke Free app via an on-screen recruitment message. This message will have a link to a screening questionnaire and consent form in Qualtrics. Potential participants will complete screening questions to verify eligibility prior to the start of the pilot trial. If determined eligible, participants will then be presented with a consent form. To complete the consent form, the participants will need to check a box that says, "Yes, I agree to participate in the study." Participants initiate contact with the study team by choosing to click on the screening questionnaire in the initial on-boarding screens.

Participants will receive 3 reminder messages via e-mail or text message, to complete the questionnaires and saliva testing.

Participants will receive compensation for participating in the study, according to the following schedule: $20 compensation for the baseline survey; $40 for the follow-up survey; $40 for the cotinine test. Details on compensation will be provided to potential participants in the consent form.

7 STUDY INTERVENTION

7.1 STUDY INTERVENTION

7.1.1 STUDY INTERVENTION DESCRIPTION

During this study, approximately one-half of study participants will receive a gamified version of the Smoke Free app. The control in this study is the "core" version of the Smoke Free app without any game component.

The core app is the lowest-cost and most popular paid version of Smoke Free. The core app includes a customizable dashboard that displays health gains and financial savings from quitting, a cravings log, a diary, a badge system, daily missions to support the quit attempt, and a Quit Coach chatbot. The missions were found to be part of a bundle of features that improve smoking abstinence at 3 months.[37] The chatbot has been found to be effective at increasing user engagement.[38]

The game intervention involves Smoke Free users maintaining a digital pet dragon, an in-game avatar that evolves during the quit attempt. The user earns points by engaging in selected in-app activities, including those in the game (e.g., playing a mini-game or feeding the dragon) and in the core Smoke Free app (e.g., reading tips or logging a diary). This design provides immediate feedback and a visual representation of a user's progress in quitting.

The intervention design was informed by principles from the fields of psychology and behavioral economics. For example, the use of frequent, salient in-game rewards is designed to counter the behavioral economic constructs of present bias and inattention to app use. The design further includes evidence-based practices from contingency management therapy, such as the use of escalating in-game rewards for abstinence with a reset point for lapses (to harness regret and loss aversion). The use of surprise gifts provides a variable reward structure designed to boost engagement and novelty.

The design was further informed by focus group discussions conducted with Smoke Free users to gather feedback on the proposed intervention.

7.1.2 ADMINISTRATION AND/OR DOSING

In this study, participants receive access to the intervention on the first day of the study if randomized to the intervention group. Participants will have access to the intervention for the full 56-63 days of the study period. At the conclusion of the study period, both the control and intervention groups will continue to have access to the same version of the Smoke Free app to which they had access during the study.

7.2 FIDELITY

7.2.1 INTERVENTIONIST TRAINING AND TRACKING

N/A

7.3 MEASURES TO MINIMIZE BIAS: RANDOMIZATION AND BLINDING

Participants will be digitally randomized in a 1:1 ratio to a core version of the Smoke Free app plus the game module or to a core version of the Smoke Free app. Allocation, concealed from investigators, will be performed using a random number generator with a random seed in the Smoke Free app platform.

Randomization will be stratified by age (<50 years old and ≥50 years old) and by smoking intensity (<5 cigarettes per day and ≥5 cigarettes per day on average), to facilitate assessment of effect modification.

The randomization will occur immediately after participants provide consent to participate in the study. Participants will not be able to navigate past an onboarding screen until they complete the screening questionnaire and are found to be eligible and provide consent to participate. Once they have provided consent, they will be able to advance past the onboarding screen and will automatically be granted access to the app version for their assigned study group.

Assessors of the follow-up cotinine test to assess smoking status will be blinded; others will not be.

7.4 STUDY INTERVENTION/EXPERIMENTAL MANIPULATION ADHERENCE

N/A

7.5 CONCOMITANT THERAPY

During the pilot trial, participants may also use alternative smoking cessation methods, including over-the-counter and prescribed medications. Use of alternative smoking cessations methods will be assessed in the follow-up questionnaire.

7.5.1 RESCUE THERAPY

N/A

8 STUDY INTERVENTION/EXPERIMENTAL MANIPULATION DISCONTINUATION AND PARTICIPANT DISCONTINUATION/WITHDRAWAL

8.1 DISCONTINUATION OF STUDY INTERVENTION/EXPERIMENTAL MANIPULATION

When a subject discontinues using the Smoke Free app, but does not drop out of the study, remaining study procedures will be completed as indicated by the study protocol. The participant is able to stop using the Smoke Free app at any time without taking any further action.

8.2 PARTICIPANT DISCONTINUATION/WITHDRAWAL FROM THE STUDY

Participants are free to withdraw from participation in the study at any time upon request. If a participant chooses to withdraw from the study, the participant can send an email to the study email address (smokefree@ucsf.edu). Instructions on withdrawal and a link to the study email account will also be provided in the help section of the Smoke Free app. If a participant withdraws, the study team will inform the Smoke Free app to stop collecting user data for study purposes. Smoke Free may continue to collect routine user data. Former participants will then be able to continue using the same version of the Smoke Free app that they received as part of the study.

The investigator will not discontinue a participant from the study for any reason.

The reason for participant discontinuation or withdrawal from the study will be recorded on the Case Report Form (CRF). Subjects who consent to participate in the study and are randomized but do not receive the study intervention may be replaced. This may occur if there is an error in the Qualtrics survey programming, the Qualtrics API, or the Smoke Free software code. Subjects who consent to participate in the study and are randomized and receive the study intervention, and subsequently withdraw, will not be replaced.

8.3 LOST TO FOLLOW-UP

A participant will be considered lost to follow-up if he or she fails to complete the follow-up questionnaire within 7 days or, if invited, the saliva cotinine test after at least 3 attempts of contact via text message.

The following actions must be taken if a participant fails to return to the clinic for a required study visit:

- Before a participant is deemed lost to follow-up, the investigator or designee will make every effort to regain contact with the participant (where possible, 3 text messages to the participant’s last known phone number). The e-mails will be sent every other day, and the text message will be sent the day after the third e-mail. These contact attempts will be documented in the participant’s study file.
- Should the participant continue to be unreachable, he or she will be considered to have withdrawn from the study with a primary reason of lost to follow-up.

9 STUDY ASSESSMENTS AND PROCEDURES

9.1 ENDPOINT AND OTHER NON-SAFETY ASSESSMENTS

Participants will be randomized in a 1:1 ratio to the core Smoke Free app plus the gamified module or to the core Smoke Free app. Individuals will be invited to participate in the pilot trial in the initial onboarding screens within the Smoke Free smartphone app, with a link to a screening questionnaire and consent form in Qualtrics. The randomization will occur immediately after participants provide consent to participate in the study. Participants will not be able to navigate past an onboarding screen in Smoke Free until they complete the screening questionnaire and are found to be eligible and provide consent to participate. Once they have provided consent, they will be able to advance past the onboarding screen and will automatically be granted access to the app version for their assigned study group.

After consenting, participants will receive an e-mail with a link to the baseline questionnaire in Qualtrics.

Participation will last until 56 days after the person’s selected quit day. During this time, participants may use the app as they wish, and the app will passively collect information on app engagement.

At the end of the 56-day period, participants will be asked to complete a follow-up questionnaire in Qualtrics. Those reporting in the follow-up questionnaire having been abstinent for 7 days will be mailed a salivary cotinine test kit to upload photos of the test results that biochemically verify smoking status.

9.2 SAFETY ASSESSMENTS

N/A

9.3 ADVERSE EVENTS AND SERIOUS ADVERSE EVENTS

9.3.1 DEFINITION OF ADVERSE EVENTS

OHRP defines an adverse event (AE) as any untoward or unfavorable medical occurrence in a human subject, including any abnormal sign (for example, abnormal physical exam or laboratory finding), symptom, or disease, temporally associated with the subject's participation in the research, whether or not considered related to the subject's participation in the research (modified from the definition of adverse events in the 1996 International Conference on Harmonization E-6 Guidelines for Good Clinical Practice).

AEs will be recorded in a spreadsheet, and the PI will monitor these events to grade severity, relationship to the study intervention, and to assess whether the nature, severity, frequency is unexpected, and thus warrants reporting as an unanticipated problem.

9.3.2 DEFINITION OF SERIOUS ADVERSE EVENTS

A serious adverse event (SAE) is one that meets one or more of the following criteria:

- Results in death.
- Is life-threatening (places the subject at immediate risk of death from the event as it occurred).
- Results in inpatient hospitalization or prolongation of existing hospitalization.
- Results in a persistent or significant disability or incapacity.
- Results in a congenital anomaly or birth defect.
- An important medical event that does not result in death, be life threatening, or require hospitalization may be considered an SAE when, based upon appropriate medical judgment, the event may jeopardize the subject and may require medical or surgical intervention to prevent one of the outcomes listed in this definition.

9.3.3 CLASSIFICATION OF AN ADVERSE EVENT

9.3.3.1 SEVERITY OF EVENT

All reportable AEs will be assessed by a study PI or co-investigator using the following guidelines to quantify intensity:

1. Mild: events require minimal or no treatment and do not interfere with the participant's daily activities.
2. Moderate: events result in a low level of inconvenience or concern with the therapeutic measures. Moderate events may cause some interference with functioning.
3. Severe: events interrupt a participant's usual daily activity and may require systemic drug therapy or other treatment. Severe events are usually incapacitating

9.3.3.2 RELATIONSHIP TO STUDY INTERVENTION/EXPERIMENTAL MANIPULATION

To assess relationship of an event to study intervention, the following guidelines are used:

1. Related (Possible, Probable, Definite)
    a. The event is known to occur with the study intervention.
    b. There is a temporal relationship between the intervention and event onset.
    c. The event abates when the intervention is discontinued.
    d. The event reappears upon a re-challenge with the intervention.
2. Not Related (Unlikely, Not Related)
    a. There is no temporal relationship between the intervention and event onset.
    b. An alternate etiology has been established.

9.3.3.3 EXPECTEDNESS

The study PI will be responsible for determining whether an AE is expected or unexpected. An AE will be considered unexpected if the nature, severity, or frequency of the event is not consistent with the risk information previously described for the intervention.

9.3.4 TIME PERIOD AND FREQUENCY FOR EVENT ASSESSMENT AND FOLLOW-UP

AEs and SAEs will be recorded in the data collection system throughout the study. Events will be followed for outcome information until resolution or stabilization.

The Study PI will record all reportable events with start dates occurring any time after informed consent is obtained until seven (for non-serious AEs) or 30 days (for SAEs) after the last day of study participation. In the 2-month follow-up questionnaire, participants will be asked to report the occurrence of AE/SAEs since study enrollment in an open-ended format.

9.3.5 ADVERSE EVENT REPORTING

In the unlikely event that an AE or SAE incident occurs during this study, any AE meeting the specified SAE criteria will be submitted within 10 workdays of the PI's awareness in the UCSF electronic IRB system to the UCSF CHR. This process applies to both initial and follow-up SAE reports.

9.3.6 SERIOUS ADVERSE EVENT REPORTING

See Section 8.3.5 above.

9.3.7 REPORTING EVENTS TO PARTICIPANTS

N/A

9.3.8 EVENTS OF SPECIAL INTEREST

N/A

9.3.9 REPORTING OF PREGNANCY

N/A

9.4 UNANTICIPATED PROBLEMS

9.4.1 DEFINITION OF UNANTICIPATED PROBLEMS

This protocol uses the definition of Unanticipated Problems as defined by the Office for Human Research Protections (OHRP). OHRP considers unanticipated problems involving risks to participants or others to include, in general, any incident, experience, or outcome that meets **all** of the following criteria:

- Unexpected in terms of nature, severity, or frequency given (a) the research procedures that are described in the protocol-related documents, such as the Institutional Review Board (IRB)-approved research protocol and informed consent document; and (b) the characteristics of the participant population being studied;
- Related or possibly related to participation in the research ("possibly related" means there is a reasonable possibility that the incident, experience, or outcome may have been caused by the procedures involved in the research); and
- Suggests that the research places participants or others at a greater risk of harm (including physical, psychological, economic, or social harm) than was previously known or recognized.

9.4.2 UNANTICIPATED PROBLEMS REPORTING

Incidents or events that meet the OHRP criteria for UPs require the creation and completion of a UP report form. OHRP recommends that PIs include the following information when reporting an AE, or any other incident, experience, or outcome as an UP to the IRB:

- Protocol identifying information: protocol title and number, PI's name, and the IRB project number
- A detailed description of the event, incident, experience, or outcome
- An explanation of the basis for determining that the event, incident, experience, or outcome represents an UP

- A description of any changes to the protocol or other corrective actions that have been taken or are proposed in response to the UP

To satisfy the requirement for prompt reporting, UPs will be reported using the following timeline:

- UPs will be reported to the UCSF IRB and to NIH National Institute on Aging and NIH National Cancer Institute within 10 working days of the PI becoming aware of the event, unless life-threatening or resulting in death in which case they will be reported within 24 hours.
- All UPs should be reported to appropriate institutional officials (as required by UCSF's written reporting procedures), the supporting agency head (or designee), and the OHRP within one month of the IRB's receipt of the report of the problem from the PI.

9.4.3 REPORTING UNANTICIPATED PROBLEMS TO PARTICIPANTS

N/A

10 STATISTICAL CONSIDERATIONS

10.1 STATISTICAL HYPOTHESES

- Primary Endpoint(s):

App opens:

- Our null hypothesis: No difference between intervention and control participants in mean number of app opens through the end of the 56-63 day program.
- Primary alternate hypothesis: Intervention and control participants differ in mean number of app opens through the end of the 56-63 day program.

Minutes per session:

- Our null hypothesis: No difference between intervention and control participants in mean number of minutes per session through the end of the 56-63 day program.
- Primary alternate hypothesis: Intervention and control participants differ in mean number of minutes per session through the end of the 56-63 day program.

- Secondary Endpoint(s):

Secondary hypotheses relate to differences between intervention and control participants in the secondary outcomes. (See Section 3.)

10.2 SAMPLE SIZE DETERMINATION

The pilot trial sample size is powered to detect meaningful differences in our primary outcome of number of times the app is opened.

As this is a pilot study, a main objective will be to provide empirical data for effect size estimation for a future full-scale RCT. Thus, our sample size is based on budget and feasible accrual during the study timeline.

With two parallel groups, the sample size calculation was conducted for one of our primary outcomes, a continuous measure of the number of times the Smoke Free app was opened through Day 56 of the study period.

We anticipate that a sample size of 500, with an assumed 70% retention, toward the high end for app-based smoking cessation trials,[19, 39] will yield a final sample size of 350 at the final assessment. We further anticipate a mean number of app opens of 30.0 per participant and a standard deviation of 45.0 in the core app, based on recent data from users of Smoke Free. A sample size of 350 participants will allow us to detect a between-group difference in app opens, one of the primary outcomes, of 13.5 at 80% power (α=0.05).

10.3 POPULATIONS FOR ANALYSES

The main analyses (i.e., for primary endpoints), unless otherwise noted, will be performed on an intent-to-treat basis that include all randomized participants.

The main analysis of smoking abstinence will be performed on a complete-case basis, using all available outcome data in the modeling with treatment condition as randomized. Sensitivity analyses will be performed 1) on an intent-to-treat basis imputing missing as smoking and 2) using multiple imputation with chained equations to handle missing data. This analytic strategy is consistent with expert recommendations and statistical practice,[40] because the assumption of missing = smoking may be biased in favor of the group with lower attrition.[41-43]

10.4 STATISTICAL ANALYSES

10.4.1 GENERAL APPROACH

The main analyses will be conducted on an intent-to-treat basis using all randomized participants. Adjusted regression models described below will adjust for a range of potential confounders, including gender, age, race/ethnicity, baseline smoking intensity, and years smoked. Multiple imputation using chained equations (MICE) for arbitrary missingness patterns[44, 45] will be used for sensitivity analyses. In further analyses, we will estimate adjusted regression models on a per-protocol basis.

10.4.2 ANALYSIS OF THE PRIMARY ENDPOINT(S)

Our key hypothesis is that the gamified app improves engagement measured by number of app opens through Day 56 following the participant's quit date. To test this, we will compare the mean number of app opens by arm, using an adjusted linear model with an indicator for treatment arm.

10.4.3 ANALYSIS OF THE SECONDARY ENDPOINT(S)

To measure retention, we will compare the proportion who complete the program by arm using an adjusted logistic model.

To measure early efficacy, we will use adjusted logistic models to compare abstinence by treatment condition for 7- and 30-day point-prevalence. Analyses for smoking abstinence will be performed on a complete-case basis, using all available data in the modeling with treatment condition as randomized. Sensitivity analyses will be performed 1) on an intent-to-treat basis imputing missing as smoking and 2) using multiple imputation with chained equations to handle missing data. This analytic strategy is consistent with expert recommendations and statistical practice,[40] because the assumption of missing = smoking may be biased in favor of the group with lower attrition.[41-43]

Post-hoc analyses will also explore the self-reported reasons un-subscribers opt out and predictors thereof.

10.4.4 SAFETY ANALYSES

N/A

10.4.5 BASELINE DESCRIPTIVE STATISTICS

We will calculate descriptive statistics for demographic and smoking variables and compare means by study condition to check equivalence. To assess engagement, retention, and abstinence, we will carefully tally and summarize these measures and test for demographic and smoking-related variables such as age that may be related to them.

10.4.6 PLANNED INTERIM ANALYSES

No interim analyses are planned.

10.4.7 SUB-GROUP ANALYSES

We will test for effect modification of engagement by examining interactions between treatment condition and selected factors, including age, race/ethnicity, household income, smoking intensity, years smoked, dual/poly tobacco use, prior video game experience, impulsivity, and sensation seeking.

10.4.8 TABULATION OF INDIVIDUAL PARTICIPANT DATA

Individual participant data will not be listed by measure or time point.

10.4.9 EXPLORATORY ANALYSES

N/A

11 SUPPORTING DOCUMENTATION AND OPERATIONAL CONSIDERATIONS

11.1 REGULATORY, ETHICAL, AND STUDY OVERSIGHT CONSIDERATIONS

11.1.1 INFORMED CONSENT PROCESS

11.1.1.1 CONSENT/ASSENT AND OTHER INFORMATIONAL DOCUMENTS PROVIDED TO PARTICIPANTS

Consent forms describing in detail the study intervention, study procedures, and risks will be sent by e-mail to the participant, and documentation of (signed) informed consent will be completed prior to starting the study intervention.

11.1.1.2 CONSENT PROCEDURES AND DOCUMENTATION

Only English speakers above 18 years of age may participate in this study. Participants will receive an online screen with the informed consent form as soon as they qualify for the study based on the eligibility screening questionnaire. Participants will be required to check a box at the end of the informed consent form to indicate "Yes, I agree to participate in the study."

11.1.2 STUDY DISCONTINUATION AND CLOSURE

This study may be temporarily suspended or prematurely terminated if there is sufficient reasonable cause. Written notification, documenting the reason for study suspension or termination, will be provided by the suspending or terminating party to study participants, investigator, funding agency, and regulatory authorities. If the study is prematurely terminated or suspended, the Principal Investigator (PI) will promptly inform study participants, the Institutional Review Board (IRB), and sponsor/funding agency and will provide the reason(s) for the termination or suspension. Study participants will be contacted, as applicable, and be informed of changes to study visit schedule.

Circumstances that may warrant termination or suspension include, but are not limited to:

- Determination of unexpected, significant, or unacceptable risk to participants

- Insufficient compliance of study staff to the protocol (ie, significant protocol violations)
- Data that are not sufficiently complete and/or evaluable
- Determination that the primary endpoint has been met
- Determination of futility

The study may resume once concerns about safety, protocol compliance, and data quality are addressed, and satisfy the funding agency, sponsor, IRB, Food and Drug Administration (FDA), or other relevant regulatory or oversight bodies.

11.1.3 CONFIDENTIALITY AND PRIVACY

During this pilot trial, data will be collected via Qualtrics, the Smoke Free app, and photographs (to verify saliva cotinine test results).

Confidentiality will be maintained via study identification (ID) codes assigned to individual participants and removal of identifying information from all transcripts, survey questionnaires, or other data records.

Only research team members and staff associated with UCSF's IRB will have access to the study data.

Participant confidentiality is strictly held in trust by the investigators and study staff.

The study protocol, documentation, data, and all other information generated will be held in strict confidence. No information concerning the study or the data will be released to any unauthorized third party without prior written approval of the PI.

23 Limited, which operates the Smoke Free app, will collect de-identified information on study participants' app usage for the 56-63 day study period. The file will be shared via Box with its TLS 1.2 encryption and linked to study data using the de-identified study identifier.

11.1.4 FUTURE USE OF STORED SPECIMENS AND DATA

N/A

11.1.5 KEY ROLES AND STUDY GOVERNANCE

**Principal Investigator**	**Study Coordinator**
Dr. Justin White, PhD, Associate Professor	Elena McGahey, BA, Study Coordinator
UCSF	UCSF
Campus Box 0936<br>490 Illinois Street, Floor 7<br>San Francisco, CA 94143	Campus Box 0936<br>490 Illinois Street, Floor 7<br>San Francisco, CA 94143
415-476-8045	415-867-5407
Justin.white@ucsf.edu	Elena.mcgahey@ucsf.edu

11.1.6 SAFETY OVERSIGHT

There is no DSMB in place for this project because it is a pilot study that it poses minimal risk to participants as determined by the UCSF IRB.

11.1.7 CLINICAL MONITORING

N/A

11.1.8 QUALITY ASSURANCE AND QUALITY CONTROL

Quality control (QC) procedures will be implemented as follows:

Source documents and the electronic data --- Data will be initially captured on source documents (see **Section 11.1.9, Data Handling and Record Keeping**) and will ultimately be entered into the study database. To ensure accuracy investigators will compare a representative sample of source data against the database, targeting key data points in that review.

Intervention Fidelity — Consistent delivery of the study interventions will be monitored throughout the intervention phase of the study by the Smoke Free team. Procedures for ensuring fidelity of intervention delivery are described in **Section 7.2.1, Interventionist Training and Tracking**.

Protocol Deviations – The study team will review protocol deviations on an ongoing basis and will implement corrective actions when the quantity or nature of deviations are deemed to be at a level of concern.

In addition, all study personnel will be appropriately trained in his/her position's duties with appropriate Human Subjects Research training. They will be provided with full information about the study such as background and significance, specific aims, milestones, study designs, procedures, and analytic plans. All team members will be encouraged to view themselves as crucial team members encouraged to comment, ask questions, and make suggestions to ensure the highest study quality; robust and rigorous data will be gathered and handled according to protocol.

The PI will ensure that this study is conducted in full conformity with the principles set forth in The Belmont Report: Ethical Principles and Guidelines for the Protection of Human Subjects of Research, as drafted by the US National Commission for the Protection of Human Subjects of Biomedical and Behavioral Research (April 18, 1979) and codified in 45 CFR Part 46 and/or the ICH E6.

Should independent monitoring become necessary, the PI will provide direct access to all trial related sites, source data/documents, and reports for the purpose of monitoring and auditing by the sponsor/funding agency, and inspection by local and regulatory authorities.

11.1.9 DATA HANDLING AND RECORD KEEPING

11.1.9.1 DATA COLLECTION AND MANAGEMENT RESPONSIBILITIES

During this pilot trial, data will be collected via Qualtrics, the Smoke Free app, and photographs (to verify cotinine test status).

Confidentiality will be maintained via study identification (ID) codes assigned to individual participants and removal of identifying information from all transcripts, survey questionnaires, or other data records.

Only bona-fide research team members and staff associated with UCSF's IRB will have access to the study data.

The study protocol, documentation, data, and all other information generated will be held in strict confidence. No information concerning the study or the data will be released to any unauthorized third party without prior written approval of the PI.

23 Limited, which operates the Smoke Free app, will collect de-identified information on study participants' app usage patterns. The file will be shared via Box with its TLS 1.2 encryption and linked to study data using the de-identified study identifier.

11.1.9.2 STUDY RECORDS RETENTION

Study documents will be retained for a minimum of 2 years after the last approval of a marketing application in an International Council on Harmonisation (ICH) region and until there are no pending or contemplated marketing applications in an ICH region or until at least 2 years have elapsed since the formal discontinuation of clinical development of the study intervention. These documents should be retained for a longer period, however, if required by local regulations. No records will be destroyed without the written consent of the sponsor/funding agency, if applicable. It is the responsibility of the sponsor/funding agency to inform the investigator when these documents no longer need to be retained.

11.1.10 PROTOCOL DEVIATIONS

This protocol defines a protocol deviation as any noncompliance with the clinical trial protocol, International Council on Harmonisation Good Clinical Practice (ICH GCP), or Manual of Procedures (MOP) requirements. The noncompliance may be either on the part of the participant, the investigators, or Smoke Free employees. As a result of deviations, corrective actions will be developed by the site and implemented promptly.

These practices are consistent with ICH GCP:

- Section 4.5 Compliance with Protocol, subsections 4.5.1, 4.5.2, and 4.5.3
- Section 5.1 Quality Assurance and Quality Control, subsection 5.1.1
- Section 5.20 Noncompliance, subsections 5.20.1, and 5.20.2.

It will be the responsibility of the PI to use continuous vigilance to identify and report deviations within 10 working days of identification of the protocol deviation, or within 10 working days of the scheduled protocol-required activity. All deviations will be addressed in study source documents, reported to NIH National Cancer Institute and NIH National Institute on Aging Program Official and to UCSF. Protocol deviations will be sent to the reviewing Institutional Review Board (IRB) per their policies. The site investigator will be responsible for knowing and adhering to the reviewing IRB requirements.

Major (reportable) protocol violations are any unapproved changes in the research study design and/or procedures that are within the investigator's control and not in accordance with the IRB-approved protocol that may affect the participant's rights, safety or well-being, or the completeness, accuracy and reliability of the study data. We **will report** all major violations to the HRPP/IRB using the Protocol Violation/Incident Report Form in iRIS and also to the NIH as described above.

Minor (non-reportable) protocol violations (also known as protocol deviations) are any unapproved changes in the research study design and/or procedures that are within the investigator's control and not in accordance with the IRB-approved protocol that **do not have a major impact** on either the participant's rights, safety or well-being, or the completeness, accuracy and reliability of the study data. We will **not report** minor protocol violations to the UCSF IRB/HRPP, but we will document them in the study files and report them to the NIH if applicable.

11.1.11 PUBLICATION AND DATA SHARING POLICY

This study will be conducted in accordance with the following publication and data sharing policies and regulations:

National Institutes of Health (NIH) Public Access Policy, which ensures that the public has access to the published results of NIH funded research. It requires scientists to submit final peer-reviewed journal manuscripts that arise from NIH funds to the digital archive PubMed Central upon acceptance for publication.

This study will comply with the NIH Data Sharing Policy and Policy on the Dissemination of NIH-Funded Clinical Trial Information and the Clinical Trials Registration and Results Information Submission rule. As such, this trial will be registered at ClinicalTrials.gov, and results from this trial will be submitted to ClinicalTrials.gov. In addition, every attempt will be made to publish results in peer-reviewed journals. Data from this study may be requested from other researchers after the completion of the primary endpoint by contacting Dr. Justin White (UCSF). Considerations for ensuring confidentiality of these shared data are described in Section 11.1.3.

11.1.12 CONFLICT OF INTEREST POLICY

The independence of this study from any actual or perceived influence, such as by the pharmaceutical industry or the tobacco industry, is critical. Therefore, any actual conflict of interest of persons who have a role in the design, conduct, analysis, publication, or any aspect of this trial will be disclosed and managed.

Furthermore, persons who have a perceived conflict of interest will be required to have such conflicts managed in a way that is appropriate to their participation in the design and conduct of this trial. The study leadership in conjunction with the NIH National Cancer Institute and the NIH National Institute on Aging has established policies and procedures for all study group members to disclose all conflicts of interest and will establish a mechanism for the management of all reported dualities of interest.

11.2 ADDITIONAL CONSIDERATIONS

N/A

11.3 ABBREVIATIONS AND SPECIAL TERMS

AE	Adverse Event
ANCOVA	Analysis of Covariance
CFR	Code of Federal Regulations
CLIA	Clinical Laboratory Improvement Amendments
CMP	Clinical Monitoring Plan
COC	Certificate of Confidentiality
CONSORT	Consolidated Standards of Reporting Trials
CRF	Case Report Form
DCC	Data Coordinating Center
DHHS	Department of Health and Human Services
DSMB	Data Safety Monitoring Board
DRE	Disease-Related Event
EC	Ethics Committee
eCRF	Electronic Case Report Forms
FDA	Food and Drug Administration
FDAAA	Food and Drug Administration Amendments Act of 2007
FFR	Federal Financial Report
GCP	Good Clinical Practice
GLP	Good Laboratory Practices
GMP	Good Manufacturing Practices
GWAS	Genome-Wide Association Studies
HIPAA	Health Insurance Portability and Accountability Act
IB	Investigator's Brochure
ICH	International Council on Harmonisation
ICMJE	International Committee of Medical Journal Editors
IDE	Investigational Device Exemption
IND	Investigational New Drug Application
IRB	Institutional Review Board
ISM	Independent Safety Monitor
ITT	Intention-To-Treat
LSMEANS	Least-squares Means
MedDRA	Medical Dictionary for Regulatory Activities

MOP	Manual of Procedures
NCT	National Clinical Trial
NIH	National Institutes of Health
NIH IC	NIH Institute or Center
OHRP	Office for Human Research Protections
PI	Principal Investigator
QA	Quality Assurance
QC	Quality Control
SAE	Serious Adverse Event
SAP	Statistical Analysis Plan
SMC	Safety Monitoring Committee
SOA	Schedule of Activities
SOC	System Organ Class
SOP	Standard Operating Procedure
UP	Unanticipated Problem
US	United States

11.4 PROTOCOL AMENDMENT HISTORY

The table below is intended to capture changes of IRB-approved versions of the protocol, including a description of the change and rationale. A ***Summary of Changes*** *table for the current amendment is located in the* ***Protocol Title Page****.*

Version	Date	Description of Change	Brief Rationale

12 REFERENCES

1. US Department of Health and Human Services. The health consequences of smoking—50 years of progress: A report of the Surgeon General. Atlanta, GA: US Department of Health and Human Services, Centers for Disease Control and Prevention, National Center for Chronic Disease Prevention and Health Promotion, Office on Smoking and Health, 2014.
2. Rafful C, García-Rodríguez O, Wang S, Secades-Villa R, Martínez-Ortega JM, Blanco C. Predictors of quit attempts and successful quit attempts in a nationally representative sample of smokers. Addict Behav. 2013;38(4):1920-3.
3. Babb S. Quitting smoking among adults—United States, 2000–2015. MMWR Morbidity and Mortality Weekly Report. 2017;65.
4. Organization. USNCIaWH. The Economics of Tobacco and Tobacco Control. In: 16-CA-8029A NCITCMNPN, editor. Bethesda, MD: U.S. Department of Health and Human Services, National Institutes of Health, National Cancer Institute; and Geneva, CH: World Health Organization; 2016.
5. Henley SJ. Vital signs: disparities in tobacco-related cancer incidence and mortality—United States, 2004–2013. MMWR Morbidity and mortality weekly report. 2016;65(44):1212-8.
6. Vidrine JI, Reitzel LR, Wetter DW. The role of tobacco in cancer health disparities. Curr Oncol Rep. 2009;11(6):475.
7. Fiscella K, Franks P, Doescher MP, Saver BG. Disparities in health care by race, ethnicity, and language among the insured: findings from a national sample. Med Care. 2002;40(1):52-9.
8. Hargraves JL, Hadley J. The contribution of insurance coverage and community resources to reducing racial/ethnic disparities in access to care. Health Serv Res. 2003;38(3):809-29.
9. Cokkinides VE, Halpern MT, Barbeau EM, Ward E, Thun MJ. Racial and ethnic disparities in smoking-cessation interventions: analysis of the 2005 National Health Interview Survey. Am J Prev Med. 2008;34(5):404-12.
10. Franks P, Fiscella K, Meldrum S. Racial disparities in the content of primary care office visits. J Gen Intern Med. 2005;20(7):599-603.
11. Abroms LC, Lee Westmaas J, Bontemps-Jones J, Ramani R, Mellerson J. A content analysis of popular smartphone apps for smoking cessation. Am J Prev Med. 2013;45(6):732--6. doi: *http://dx.doi.org/10.1016/j.amepre.2013.07.008*.
12. Pew Research Center. Mobile Fact Sheet2018. Available from: *http://www.pewinternet.org/fact-sheet/mobile/*.
13. Haskins BL, Lesperance D, Gibbons P, Boudreaux ED. A systematic review of smartphone applications for smoking cessation. Transl Behav Med. 2017;7(2):292-9.
14. Bricker JB, Mull KE, Kientz JA, Vilardaga R, Mercer LD, Akioka KJ, Heffner JL. Randomized, controlled pilot trial of a smartphone app for smoking cessation using acceptance and commitment therapy. Drug Alcohol Depend. 2014;143(0):87 - 94. doi: *http://dx.doi.org/10.1016/j.drugalcdep.2014.07.006*.
15. Ubhi HK, Michie S, Kotz D, Wong WC, West R. A mobile app to aid smoking cessation: preliminary evaluation of SmokeFree28. J Med Internet Res. 2015;17(1).

16. Bricker JB, Copeland W, Mull KE, Zeng EY, Watson NL, Akioka KJ, Heffner JL. Single-arm trial of the second version of an acceptance & commitment therapy smartphone application for smoking cessation. Drug Alcohol Depend. 2017;170:37-42.
17. Iacoviello BM, Steinerman JR, Klein DB, Silver TL, Berger AG, Luo SX, Schork NJ. Clickotine, a personalized smartphone app for smoking cessation: initial evaluation. JMIR mHealth uHealth. 2017;5(4).
18. BinDhim NF, McGeechan K, Trevena L. Smartphone Smoking Cessation Application (SSC App) trial: a multicountry double-blind automated randomised controlled trial of a smoking cessation decision-aid 'app'. BMJ open. 2018;8(1):e017105.
19. Krebs P, Burkhalter J, Fiske J, Snow H, Schofield E, Iocolano M, Borderud S, Ostroff JS. The QuitIT Coping Skills Game for Promoting Tobacco Cessation Among Smokers Diagnosed With Cancer: Pilot Randomized Controlled Trial. JMIR mHealth uHealth. 2019;7(1):e10071. Epub 10.01.2019. doi: 10.2196/10071. PubMed PMID: 30632971.
20. Marler JD, Fujii CA, Utley DS, Tesfamariam LJ, Galanko JA, Patrick H. Initial Assessment of a Comprehensive Digital Smoking Cessation Program That Incorporates a Mobile App, Breath Sensor, and Coaching: Cohort Study. JMIR mHealth uHealth. 2019;7(2):e12609. Epub 04.02.2019. doi: 10.2196/12609. PubMed PMID: 30670372.
21. van den Brand FA, Nagelhout GE, Winkens B, Chavannes NH, van Schayck OC. Effect of a workplace-based group training programme combined with financial incentives on smoking cessation: A cluster-randomised controlled trial. The Lancet Public Health. 2018. doi: *http://dx.doi.org/10.1016/S2468-2667(18)30185-3*.
22. Cole-Lewis H, Augustson E, Sanders A, Schwarz M, Geng Y, Coa K, Hunt Y. Analysing user-reported data for enhancement of SmokefreeTXT: a national text message smoking cessation intervention. Tob Control. 2017;26:683-9.
23. Consumer Health Information Corporation. Motivating patients to use smartphone health apps 2011 [updated April 25, 2011May 20, 2017]. Available from: *http://www.prweb.com/releases/2011/04/prweb5268884.htm*.
24. Cafazzo AJ, Casselman M, Hamming N, Katzman KD, Palmert RM. Design of an mHealth app for the self-management of adolescent type 1 diabetes: A pilot study. J Med Internet Res. 2012;14(3):e70. doi: 10.2196/jmir.2058.
25. Primack BA, Carroll MV, McNamara M, Klem ML, King B, Rich M, Chan CW, Nayak S. Role of video games in improving health-related outcomes. Am J Prev Med. 2012;42(6):630--8.
26. King D, Greaves F, Exeter C, Darzi A. `Gamification': Influencing health behaviours with games. J R Soc Med. 2013;106(3):76-8. doi: 10.1177/0141076813480996.
27. Richardson A, Graham AL, Cobb N, Xiao H, Mushro A, Abrams D, Vallone D. Engagement promotes abstinence in a web-based cessation intervention: cohort study. J Med Internet Res. 2013;15(1):e14. Epub 2013/01/29. doi: 10.2196/jmir.2277. PubMed PMID: 23353649; PMCID: PMC3636070.
28. Berry K, Salter A, Morris R, James S, Bucci S. Assessing Therapeutic Alliance in the Context of mHealth Interventions for Mental Health Problems: Development of the Mobile Agnew Relationship Measure (mARM) Questionnaire. J Med Internet Res. 2018;20(4):e90. Epub 19.04.2018. doi: 10.2196/jmir.8252. PubMed PMID: 29674307.
29. D'Alfonso S, Lederman R, Bucci S, Berry K. The Digital Therapeutic Alliance and Human-Computer Interaction. JMIR Mental Health. 2020;7(12):e21895.

30. Looyestyn J, Kernot J, Boshoff K, Ryan J, Edney S, Maher C. Does gamification increase engagement with online programs? A systematic review. PLoS One. 2017;12(3):e0173403. Epub 2017/04/01. doi: 10.1371/journal.pone.0173403. PubMed PMID: 28362821; PMCID: PMC5376078.
31. Theng YL, Lee JW, Patinadan PV, Foo SS. The Use of Videogames, Gamification, and Virtual Environments in the Self-Management of Diabetes: A Systematic Review of Evidence. Games Health J. 2015;4(5):352-61. Epub 2015/08/20. doi: 10.1089/g4h.2014.0114. PubMed PMID: 26287926.
32. Patel MS, Benjamin EJ, Volpp KG, Fox CS, Small DS, Massaro JM, Lee JJ, Hilbert V, Valentino M, Taylor DH. Effect of a game-based intervention designed to enhance social incentives to increase physical activity among families: the BE FIT randomized clinical trial. JAMA Intern Med. 2017;177(11):1586-93.
33. Roepke AM, Jaffee SR, Riffle OM, McGonigal J, Broome R, Maxwell B. Randomized controlled trial of SuperBetter, a smartphone-based/Internet-based self-help tool to reduce depressive symptoms. Games for health journal. 2015;4(3):235-46.
34. El-Hilly AA, Iqbal SS, Ahmed M, Sherwani Y, Muntasir M, Siddiqui S, Al-Fagih Z, Usmani O, Eisingerich AB. Game on? Smoking cessation through the gamification of mHealth: A longitudinal qualitative study. JMIR Serious Games. 2016;4(2).
35. Raiff BR, Fortugno N, Scherlis DR, Rapoza D. A Mobile Game to Support Smoking Cessation: Prototype Assessment. JMIR Serious Games. 2018;6(2):e11. Epub 07.06.2018. doi: 10.2196/games.9599.
36. Bricker JB, Watson NL, Mull KE, Sullivan BM, Heffner JL. Efficacy of Smartphone Applications for Smoking Cessation: A Randomized Clinical Trial. JAMA Intern Med. 2020;180(11):1472-80. doi: 10.1001/jamainternmed.2020.4055.
37. Crane D, Ubhi HK, Brown J, West R. Relative effectiveness of a full versus reduced version of the `Smoke Free’ mobile application for smoking cessation: an exploratory randomised controlled trial [version 2; peer review: 2 approved]. F1000Research. 2019;7(1524). doi: 10.12688/f1000research.16148.2.
38. Perski O, Crane D, Beard E, Brown J. Does the addition of a supportive chatbot promote user engagement with a smoking cessation app? An experimental study. DIGITAL HEALTH. 2019;5:2055207619880676. doi: 10.1177/2055207619880676. PubMed PMID: 31620306.
39. Baskerville NB, Struik LL, Guindon GE, Norman CD, Whittaker R, Burns C, Hammond D, Dash D, Brown KS. Effect of a Mobile Phone Intervention on Quitting Smoking in a Young Adult Population of Smokers: Randomized Controlled Trial. JMIR mHealth uHealth. 2018;6(10):e10893-e.
40. Hall SM, Delucchi KL, Tsoh JY, Velicer WF, Kahler CW, Moore JR, Hedeker D, Niaura R. Statistical analysis of randomized trials in tobacco treatment: Longitudinal designs with dichotomous outcome. Nicotine Tob Res. 2001;3(3):193-202.
41. Hedeker D, Mermelstein RJ, Demirtas H. Analysis of binary outcomes with missing data: missing = smoking, last observation carried forward, and a little multiple imputation. Addiction. 2007;102(10):1564--73. doi: 10.1111/j.1360-0443.2007.01946.x.
42. Nelson DB, Partin MR, Fu SS, Joseph AM, An LC. Why assigning ongoing tobacco use is not necessarily a conservative approach to handling missing tobacco cessation outcomes. Nicotine Tob Res. 2009;11(1):77-83.

43. Blankers M, Smit ES, van der Pol P, de Vries H, Hoving C, van Laar M. The Missing=Smoking Assumption: A Fallacy in Internet-Based Smoking Cessation Trials? Nicotine Tob Res. 2015;18(1):25-33. doi: 10.1093/ntr/ntv055.
44. Raghunathan TE, Lepkowski JM, Van Hoewyk J, Solenberger P. A multivariate technique for multiply imputing missing values using a sequence of regression models. Survey methodology. 2001;27(1):85-96.
45. Van Buuren S. Multiple imputation of discrete and continuous data by fully conditional specification. Stat Methods Med Res. 2007;16(3):219-42.
