# Supplementary material for: Evaluating the Impact of a Game (Inner Dragon) on User Engagement Within a Leading Smartphone App for Smoking Cessation: Randomized Controlled Trial
Source: J Med Internet Res. 2024 Oct 30;26:e57839. doi: 10.2196/57839 (PMC11561441; doi:10.2196/57839)
Supplement: Multimedia Appendix 3 [file jmir_v26i1e57839_app3.docx]

**Supplementary Appendix**

Supplement to: “Evaluating the Impact of a Game (Inner Dragon) on User Engagement Within a Leading Smartphone App for Smoking Cessation: A Randomized Controlled Trial.”

**Supplemental Tables and Figures**

[**Table S1.** Descriptive statistics with test of the difference in means by arm 2](#_Toc164332281)

[**Table S2.** Descriptive statistics for game feature use 3](#_Toc164332282)

[**Table S3.** Covariate-adjusted differences in outcomes by study group 4](#_Toc164332283)

[**Table S4.** Intent-to-treat abstinence estimates reported in previous RCTs of app-based smoking cessation interventions with follow-up of 1 to 3 months 5](#_Toc164332284)

[**Figure S1.** Sensitivity of changes in abstinence to assumptions about missingness using pattern mixture model 5](#_Toc164262466)

[**Figure S2.** Satisfaction with Smoke Free by study group 6](#_Toc164262467)

[**Figure S3.** Cumulative distribution of the number of total sessions and sessions with game use 7](#_Toc164262468)

[**Figure S4.** Distribution of game feature use 8](#_Toc164262469)

[**Figure S5.** Correlation between total number of app sessions and Inner Dragon features 9](#_Toc164262470)

[**Figure S6.** Subgroup estimates of differences in primary outcomes by study group 10](#_Toc164262471)

**Table S1.** Descriptive statistics with test of the difference in means by arm

|  | Mean | |  |  |
| --- | --- | --- | --- | --- |
|  | Control  (n = 238) | Treated  (n = 241) | Difference  in means | P-value |
| **Panel A. Demographics** |  |  |  |  |
| Age, in years | 37.4 | 38.5 | 1.0 | 0.32 |
| Gender, % |  |  |  |  |
| Female | 76.1 | 73.4 | -2.6 | 0.51 |
| Male | 23.1 | 24.5 | 1.4 | 0.73 |
| Non-binary/other | 0.8 | 2.1 | 1.2 | 0.26 |
| Race and ethnicity, % |  |  |  |  |
| Non-Hispanic White | 76.5 | 77.2 | 0.7 | 0.85 |
| Non-Hispanic Black | 10.9 | 9.1 | -1.8 | 0.51 |
| Hispanic | 3.8 | 7.9 | 4.1 | 0.06 |
| Other | 8.8 | 5.8 | -3.0 | 0.21 |
| Household income, % |  |  |  |  |
| Less than $20,000 | 14.7 | 14.5 | -0.2 | 0.95 |
| Between $20,000 and $40,000 | 27.3 | 26.1 | -1.2 | 0.77 |
| Between 40,000 and $60,000 | 21.4 | 21.2 | -0.3 | 0.94 |
| Between 60,000 and $80,000 | 10.1 | 13.3 | 3.2 | 0.28 |
| Between 80,000 and $100,000 | 7.6 | 8.7 | 1.2 | 0.65 |
| More than $100,000 | 18.9 | 16.2 | -2.7 | 0.43 |
| Education, % |  |  |  |  |
| High school diploma or less | 23.1 | 18.7 | -4.4 | 0.23 |
| Some college or technical school | 35.3 | 38.6 | 3.3 | 0.46 |
| Bachelor's or associate degree | 28.6 | 34.4 | 5.9 | 0.17 |
| Graduate degree | 13.0 | 8.3 | -4.7 | 0.09 |
|  |  |  |  |  |
| **Panel B. Smoking characteristics** |  |  |  |  |
| Cigarettes per day | 15.3 | 15.8 | 0.5 | 0.47 |
| Fagerström Test | 4.9 | 5.2 | 0.3 | 0.20 |
| Past quit attempts | 6.5 | 6.7 | 0.2 | 0.82 |
| Years since initiated | 19.5 | 20.6 | 1.0 | 0.35 |
| Used ENDS in last 30 days, % | 25.6 | 23.7 | -2.0 | 0.62 |
| Used NRT in last 30 days, % | 18.1 | 23.2 | 5.2 | 0.16 |
|  |  |  |  |  |
| **Panel C. Other** |  |  |  |  |
| Frequency played video games, % |  |  |  |  |
| Not at all | 24.8 | 23.7 | -1.1 | 0.77 |
| Less than once a month | 10.5 | 14.9 | 4.4 | 0.15 |
| At least monthly but not weekly | 10.5 | 10.8 | 0.3 | 0.92 |
| At least weekly but not every day | 16.8 | 20.3 | 3.5 | 0.32 |
| Every day | 37.4 | 30.3 | -7.1 | 0.10 |

Note: The p-value is from a *t*-test of the difference in means.

**Table S2.** Descriptive statistics for game feature use

| Game feature | Mean (SD) | Median (IQR) |
| --- | --- | --- |
| Breathing exercise | 3.6 (8.2) | 1 (0-4) |
| Cleaned dragon | 4.8 (10.4) | 1 (0-5) |
| Fed dragon | 4.9 (10.6) | 1 (0-5) |
| Memory minigame | 2.5 (4.9) | 1 (0-3) |
| Customization changes | 2.4 (4.4) | 1 (0-2) |
| Opened guide | 7.7 (12.5) | 3 (1-8) |
| Visited Dragon Park | 3.0 (4.7) | 1 (0-4) |
| Gifts received | 4.7 (7.7) | 1 (0-6) |
| Read user profiles | 2.2 (4.1) | 1 (0-2) |
| Sent messages | 0.9 (2.5) | 0 (0-1) |
| Times awarded experience points | 45.2 (104.8) | 9 (0-43) |

Note: Inner Dragon game features were available to those in the treated group only.

**Table S3.** Covariate-adjusted differences in outcomes by study group

|  |  | Coef. | 95% CI | p-value | N |
| --- | --- | --- | --- | --- | --- |
| *Panel A. User engagement* | |  |  |  |  |
| No. app sessions | | 4.57 | (-2.18,11.31) | 0.18 | 479 |
| Minutes per session | | 0.83 | (-0.19, 1.85) | 0.11 | 479 |
| No. days with a session | | 2.15 | (-0.44, 4.74) | 0.10 | 479 |
| Index of core feature use | | 6.60 | (-0.39,13.59) | 0.06 | 479 |
| Use of core features | |  |  |  |  |
| No. cravings reported | | 0.85 | (0.02, 1.69) | 0.05 | 479 |
| No. diary entries | | 2.72 | (0.07, 5.36) | 0.04 | 479 |
| No. missions completed | | 0.97 | (-0.77, 2.71) | 0.28 | 479 |
| No. chatbot sessions | | 1.42 | (-0.53, 3.38) | 0.15 | 479 |
|  | |  |  |  |  |
| *Panel B. Point-prevalence abstinence* | |  |  |  |  |
| 7-day point-prevalence abstinence at 2 months, in pp | |  |  |  |  |
| Self-reported, missing = smoking | | -2.36 | (-11.09, 6.38) | 0.60 | 479 |
| Self-reported, complete cases | | -12.40 | (-22.68, -2.11) | 0.02 | 292 |
| Verified, missing = smoking | | -2.71 | (-10.01, 4.60) | 0.47 | 479 |
| Verified, complete cases | | -7.20 | (-21.81, 7.40) | 0.33 | 128 |
| 30-day point-prevalence abstinence at 2 months, in pp | |  |  |  |  |
| Self-reported, missing = smoking | | 1.44 | (-5.97, 8.85) | 0.70 | 479 |
| Self-reported, complete cases | | -2.51 | (-13.40, 8.38) | 0.65 | 292 |
| Mean repeated 1-day abstinence, in pp | |  |  |  |  |
| Self-reported, missing = smoking | | 4.45 | (0.18, 8.72) | 0.04 | 4.45 |
| Self-reported, complete cases | | 10.06 | (1.58,18.54) | 0.02 | 10.06 |
|  | |  |  |  |  |
| *Panel C. Satisfaction and motivation* | |  |  |  |  |
| Satisfaction with app | | 0.04 | (-0.22, 0.31) | 0.75 | 271 |
| Recommend app to friends | | -0.12 | (-0.38, 0.15) | 0.39 | 271 |
| Motivation to (stay) quit | | -0.12 | (-0.64, 0.39) | 0.63 | 144 |
| Digital therapeutic alliance index | | -0.11 | (-0.68, 0.47) | 0.71 | 268 |

Note: The coefficient denotes the difference between the treated and control groups. Regressions are adjusted for the demographic, smoking history, and gaming experience characteristics listed in Table 1. Linear regressions are used for continuous outcomes and logistic regressions, with coefficients expressed as risk differences, for binary outcomes.

**Table S4.** Intent-to-treat abstinence estimates reported in previous RCTs of app-based smoking cessation interventions with follow-up of 1 to 3 months

|  | **First author** | **Year** | **Treated arm** | **Control arm** | **No. Treated** | **No. Control** | **N** | **Type** | **Abstin. period, days** | **Follow-up, mos.** | **No. Abstain, Treated** | **No. Abstain, Control** | **Abst. %, T** | **Abst. %, C** | **ITT Effect** |
| --- | --- | --- | --- | --- | --- | --- | --- | --- | --- | --- | --- | --- | --- | --- | --- |
| 1 | Baskerville [1] | 2018 | Crush the Crave | Self-help guide | 820 | 779 | 1599 | Self-report | 7 | 3 | 105 | 107 | 12.8% | 13.7% | -0.9 |
| 2 | BinDhim [2] | 2018 | SSC App | Info-only app | 342 | 342 | 684 | Self-report | 90 | 3 | 59 | 27 | 17.3% | 7.9% | 9.4 |
| 3 | Bricker [3] | 2014 | SmartQuit | QuitGuide | 98 | 98 | 196 | Self-report | 30 | 2 | 10 | 7 | 10.2% | 7.1% | 3.1 |
| 4 | Bricker [4] | 2020 | ICanQuit | QuitGuide | 1214 | 1201 | 2415 | Self-report | 7 | 3 | 285 | 168 | 23.5% | 14.0% | 9.5 |
| 5 | Crane [5] | 2019 | Smoke Free - full | Smoke Free - lite | 14228 | 13884 | 28112 | Self-report | 90 | 3 | 234 | 124 | 1.6% | 0.9% | 0.8 |
| 6 | Goldenhersch [6] | 2020 | Mindcotine | Peer-to-peer support | 60 | 60 | 120 | Self-report | 1 | 3 | 14 | 3 | 23.3% | 5.0% | 18.3 |
| 7 | Herbec [7] | 2019 | NRT2Quit - full | NRT2Quit - minimal | 16 | 25 | 41 | Saliva | 28 | 2 | 4 | 2 | 25.0% | 8.0% | 17.0 |
| 8 | Hertzberg [8] | 2013 | mCM | Non-contingent CM | 11 | 11 | 22 | CO | 7 | 1 | 9 | 5 | 81.8% | 45.5% | 36.4 |
| 9 | Jackson [9] | 2023 | Smoke Free | None | 1564 | 1579 | 3143 | Self-report | 90 | 3 | 45 | 54 | 2.9% | 3.4% | -0.5 |
| 10 | Krebs [10] | 2019 | QuitIT game | Telecounsel., Rx | 18 | 20 | 38 | CO | 7 | 1 | 4 | 2 | 22.2% | 10.0% | 12.2 |
| 11 | Krishnan [11] | 2019 | COach2Quit | Brief advice | 50 | 52 | 102 | CO | 7 | 1 | 9 | 2 | 18.0% | 3.8% | 14.2 |
| 12 | Masaki [12] | 2020 | CureApp + CO checker | CureApp - reduced | 285 | 287 | 572 | CO | 90 | 3 | 215 | 190 | 75.4% | 66.2% | 10.8 |
| 13 | Pallejà-Millán [13] | 2020 | Tobbstop | Information | 284 | 318 | 602 | Self-report | 90 | 3 | 72 | 13 | 25.4% | 4.1% | 21.3 |
| 14 | Webb [14] | 2020 | Quit Genius | Brief advice | 265 | 265 | 530 | Self-report | 7 | 1 | 118 | 75 | 44.5% | 28.3% | 16.2 |

Note: This table summarizes RCTs of app-based smoking cessation interventions. Studies were identified through prior systematic reviews and meta-analyses [15-22], online searches of studies citing those articles, and knowledge of the authors. Sample sizes, number and percent abstaining by arm, and intent-to-treat effect were calculated by the authors, when not directly reported.

**Figure S1.** Sensitivity of changes in abstinence to assumptions about missingness using pattern mixture model

Note: This figure shows the estimated treatment effect (equal to treated group minus control group) for self-reported 7-day point-prevalence abstinence from a pattern-mixture model that varies the informative missingness odds ratio (exp(δ)), or IMOR. Following the procedure by White et al. (2011, *BMJ*), the IMOR is the odds ratio between the outcome and an indicator for missingness, adjusting for covariates. The base value of exp(δ) of 0 corresponds to missingness at random, equivalent to an assumption of missing = smoking. The data series in blue varies exp(δ) for individuals in the treated group while assuming exp(δ) = 0 for the control group. The data series in green varies exp(δ) for individuals in the control group while assuming exp(δ) = 0 for the treated group. The data series in red varies exp(δ) for all individuals in both groups.

**Figure S2.** Satisfaction with Smoke Free by study group

1. Satisfaction with Smoke Free by study group

1. Satisfaction with Inner Dragon in treated group

Note: Inset labels indicate the percentage of participants selecting that option.

**Figure S3.** Cumulative distribution of the number of total sessions and sessions with game use

Note: Panel A shows the cumulative distribution function of the total number of sessions. Panel B shows the number of sessions with game use (treated group only). For example, in Panel A, the 75^th^ percentile of treated participants had 39 sessions, and the 75^th^ percentile of control participants had 30 sessions.

**Figure S4.** Distribution of game feature use

Note: Each panel shows the cumulative distribution function of the total number of sessions (Panel A-G, I-J) or total number of events (Panels H and K) with different types of game events. For example, in Panel A, the 75^th^ percentile of treated participants had 4 sessions in which the user completed a breathing exercise.

**Figure S5.** Correlation between total number of app sessions and Inner Dragon features

Note: This figure shows a heatmap of the pairwise correlation matrix between total number of app sessions (the primary user engagement outcome), inscribed in the blue box, and selected features of the Inner Dragon game module. The figure shows a moderate (correlation 0.4 to 0.6) or strong (0.6 to 0.8) positive association between most game features and total number of app sessions, as well as a strong or very strong (0.8 to 1.0) positive association between the features with each other. All correlation coefficients are statistically significant at the 0.01 level.

**Figure S6.** Subgroup estimates of differences in primary outcomes by study group

1. Difference in sessions per user by study group

1. Difference in mean minutes per session by study group

Note: Each figure is based on a series of unadjusted stratified regressions in which each row shows the treatment effect from a single regression. The row with the “overall” estimates corresponds to the estimate for the full sample.

**References**

1. Baskerville NB, Struik LL, Guindon GE, Norman CD, Whittaker R, Burns C, et al. Effect of a Mobile Phone Intervention on Quitting Smoking in a Young Adult Population of Smokers: Randomized Controlled Trial. JMIR mHealth uHealth. 2018;6(10):e10893-e.

2. BinDhim NF, McGeechan K, Trevena L. Smartphone Smoking Cessation Application (SSC App) trial: a multicountry double-blind automated randomised controlled trial of a smoking cessation decision-aid ‘app’. BMJ Open. 2018;8(1):e017105.

3. Bricker JB, Mull KE, Kientz JA, Vilardaga R, Mercer LD, Akioka KJ, Heffner JL. Randomized, controlled pilot trial of a smartphone app for smoking cessation using acceptance and commitment therapy. Drug Alcohol Depend. 2014;143(0):87 - 94. doi: <http://dx.doi.org/10.1016/j.drugalcdep.2014.07.006>.

4. Bricker JB, Watson NL, Mull KE, Sullivan BM, Heffner JL. Efficacy of Smartphone Applications for Smoking Cessation: A Randomized Clinical Trial. JAMA Intern Med. 2020;180(11):1472-80. doi: 10.1001/jamainternmed.2020.4055.

5. Crane D, Ubhi HK, Brown J, West R. Relative effectiveness of a full versus reduced version of the `Smoke Free’ mobile application for smoking cessation: an exploratory randomised controlled trial. F1000Research. 2019;7(1524). doi: 10.12688/f1000research.16148.2.

6. Goldenhersch E, Thrul J, Ungaretti J, Rosencovich N, Waitman C, Ceberio MR. Virtual Reality Smartphone-Based Intervention for Smoking Cessation: Pilot Randomized Controlled Trial on Initial Clinical Efficacy and Adherence. J Med Internet Res. 2020 Jul 29;22(7):e17571. PMID: 32723722. doi: 10.2196/17571.

7. Herbec A, Brown J, Shahab L, West R, Raupach T. Pragmatic randomised trial of a smartphone app (NRT2Quit) to improve effectiveness of nicotine replacement therapy in a quit attempt by improving medication adherence: results of a prematurely terminated study. Trials. 2019 Sep 2;20(1):547. PMID: 31477166. doi: 10.1186/s13063-019-3645-4.

8. Hertzberg JS, Carpenter VL, Kirby AC, Calhoun PS, Moore SD, Dennis MF, et al. Mobile Contingency Management as an Adjunctive Smoking Cessation Treatment for Smokers With Posttraumatic Stress Disorder. Nicotine Tob Res. 2013;15(11):1934-8. doi: 10.1093/ntr/ntt060.

9. Jackson SE, Kale D, Beard E, Perski O, West R, Brown J. Effectiveness of the offer of the Smoke Free smartphone application compared with no intervention for smoking cessation: A pragmatic randomised controlled trial. medRxiv2023. p. 2023.01.12.23284463.

10. Krebs P, Burkhalter J, Fiske J, Snow H, Schofield E, Iocolano M, et al. The QuitIT Coping Skills Game for Promoting Tobacco Cessation Among Smokers Diagnosed With Cancer: Pilot Randomized Controlled Trial. JMIR mHealth uHealth. 2019;7(1):e10071. PMID: 30632971. doi: 10.2196/10071.

11. Krishnan N, Elf JL, Chon S, Golub JE. COach2Quit: a pilot randomized controlled trial of a personal carbon monoxide monitor for smoking cessation. Nicotine Tob Res. 2019;21(11):1573-7.

12. Masaki K, Tateno H, Nomura A, Muto T, Suzuki S, Satake K, et al. A randomized controlled trial of a smoking cessation smartphone application with a carbon monoxide checker. npj Digital Medicine. 2020 2020/03/12;3(1):35. doi: 10.1038/s41746-020-0243-5.

13. Pallejà-Millán M, Rey-Reñones C, Barrera Uriarte ML, Granado-Font E, Basora J, Flores-Mateo G, Duch J. Evaluation of the Tobbstop Mobile App for Smoking Cessation: Cluster Randomized Controlled Clinical Trial. JMIR mHealth uHealth. 2020;8(6):e15951. PMID: 32589153. doi: 10.2196/15951.

14. Webb J, Peerbux S, Smittenaar P, Siddiqui S, Sherwani Y, Ahmed M, et al. Preliminary Outcomes of a Digital Therapeutic Intervention for Smoking Cessation in Adult Smokers: Randomized Controlled Trial. JMIR Ment Health. 2020;7(10):e22833. PMID: 33021488. doi: 10.2196/22833.

15. Whittaker R, McRobbie H, Bullen C, Rodgers A, Gu Y, Dobson R. Mobile phone text messaging and app-based interventions for smoking cessation. Cochrane Database Syst Rev. 2019 Oct 22;10(10):Cd006611. PMID: 31638271. doi: 10.1002/14651858.CD006611.pub5.

16. Barnett A, Ding H, Hay KE, Yang IA, Bowman RV, Fong KM, Marshall HM. The effectiveness of smartphone applications to aid smoking cessation: A meta-analysis. Clinical eHealth. 2020 2020/01/01/;3:69-81. doi: <https://doi.org/10.1016/j.ceh.2020.09.001>.

17. Chu KH, Matheny SJ, Escobar-Viera CG, Wessel C, Notier AE, Davis EM. Smartphone health apps for tobacco cessation: A systematic review. Addict Behav. 2021 Jan;112:106616. PMID: 32932102. doi: 10.1016/j.addbeh.2020.106616.

18. Cobos-Campos R, de Lafuente AS, Apiñaniz A, Parraza N, Llanos IP, Orive G. Effectiveness of mobile applications to quit smoking: Systematic review and meta-analysis. Tob Prev Cessat. 2020;6:62. PMID: 33241162. doi: 10.18332/tpc/127770.

19. Haskins BL, Lesperance D, Gibbons P, Boudreaux ED. A systematic review of smartphone applications for smoking cessation. Transl Behav Med. 2017;7(2):292-9.

20. Regmi K, Kassim N, Ahmad N, Tuah NA. Effectiveness of Mobile Apps for Smoking Cessation: A Review. Tob Prev Cessat. 2017;3:12. PMID: 32432186. doi: 10.18332/tpc/70088.

21. Staiger PK, O'Donnell R, Liknaitzky P, Bush R, Milward J. Mobile Apps to Reduce Tobacco, Alcohol, and Illicit Drug Use: Systematic Review of the First Decade. J Med Internet Res. 2020 Nov 24;22(11):e17156. PMID: 33231555. doi: 10.2196/17156.

22. Ybarra ML, Jiang Y, Free C, Abroms LC, Whittaker R. Participant-level meta-analysis of mobile phone-based interventions for smoking cessation across different countries. Prev Med. 2016 Aug;89:90-7. PMID: 27154349. doi: 10.1016/j.ypmed.2016.05.002.
